# Metabolic profiling of liver and faeces in mice infected with echinococcosis

**DOI:** 10.1186/s13071-021-04807-1

**Published:** 2021-06-14

**Authors:** Mingxing Zhu, Xiancai Du, Hongxia Xu, Songhao Yang, Chan Wang, Yazhou Zhu, Tingrui Zhang, Wei Zhao

**Affiliations:** 1 Center of Scientific Technology of Ningxia Medical University, Yinchuan, 750004 Ningxia Hui Autonomous Region People’s Republic of China; 2 Key Laboratory of Hydatid Disease of Ningxia Medical University, Yinchuan, 750004 Ningxia Hui Autonomous Region People’s Republic of China; 3 School of Basic Medical Science of Ningxia Medical University, Yinchuan, 750004 Ningxia Hui Autonomous Region People’s Republic of China

**Keywords:** Echinococcosis, Metabolomics, Metabolites, Liquid chromatography coupled with tandem mass spectrometry (LC–MS/MS)

## Abstract

**Background:**

Echinococcosis is a severe zoonotic parasitic disease which severely affects the health of the hosts. The diagnosis of echinococcosis depends mainly on imaging examination. However, the patient is often in the late stage of the disease when the symptoms appear, thus limiting the early diagnosis of echinococcosis. The treatment and prognosis of the patients are hampered because of long-term asymptomatic latency. Metabolomics is a new discipline developed in the late 1990s. It reflects a series of biological responses in pathophysiological processes by demonstrating the changes in metabolism under the influence of internal and external factors. When the organism is invaded by pathogens, the alteration in the characteristics of metabolites in cells becomes extremely sensitive. Here, we used a metabolomics approach involving liquid chromatography coupled with tandem mass spectrometry (LC–MS/MS) to determine the molecular mechanism of cystic echinococcosis (CE) and to develop an effective method for CE diagnosis.

**Methods:**

Twenty 8-week-old female BALB/c mice were divided into normal and *Echinococcus granulosus* infection groups. To develop the *E. granulosus* infection model, mice were infected with protoscoleces. Six weeks later, the abdomens of the mice showed significant bulging. An LC–MS/MS system-based metabolomics approach was used to analyse the liver and faeces to reveal the metabolic profiles of mice with echinococcosis.

**Results:**

We found that the metabolism of nucleotides, alkaloids, amino acids, amides, and organic acids in mice is closely interrelated with *E. granulosus* infection. In the liver, the metabolic pathways of tyrosine and tryptophan biosynthesis; phenylalanine, valine, leucine and isoleucine biosynthesis; and phenylalanine metabolism were notably associated with the occurrence and development of hydatid disease, and in the faeces, pantothenate and CoA biosynthesis are thought to be closely associated with the development of CE.

**Conclusion:**

The metabolomics approach used in this study provides a reference for a highly sensitive and specific diagnostic and screening method for echinococcosis.

**Graphic Abstract:**

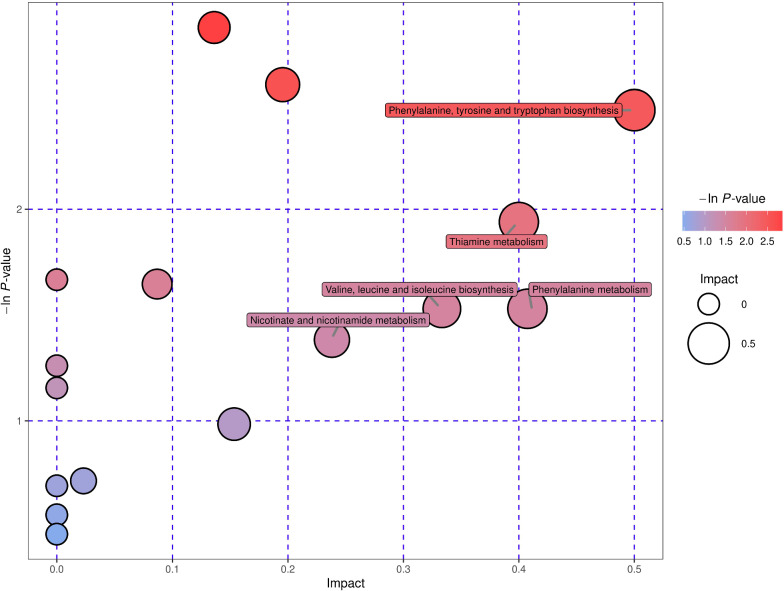

**Supplementary Information:**

The online version contains supplementary material available at 10.1186/s13071-021-04807-1.

## Background

Cystic echinococcosis (CE) or hydatidosis is a type of near-cosmopolitan zoonosis caused by *Echinococcus granulosus *sensu lato [1, 2]. The life cycle of *E. granulosus* involves two mammalian hosts. For adult tapeworms, carnivores (canines and cats) are the definitive hosts, while ungulates and rodents are intermediate hosts [3]. CE is prevalent in Western China, South America, Central Asia, the Mediterranean, and East Africa [4]. The main risk factors are close contact with dogs and livestock [5–8]. Humans are not usually directly involved in the spread of CE. However, humans or intermediate hosts accidentally ingest eggs, which are then incubated in the intestines and release oncospheres [9]. The oncospheres are transported to the liver through the portal vein and lymphatic vessels where they settle and develop into larvae (hydatid cysts), partly reaching the lungs and rarely reaching the brains, bones, or any other organ of a human or intermediate host [2, 10]. The growth of CE cysts is very slow [11]. There are no significant symptoms in the early stage of CE, with more than half of cysts expressing no change in size in 10–15 years [12]. Clinical symptoms such as epigastric discomfort or loss of appetite commonly appear when the cyst becomes more than 10 cm long, and gradually develop, causing damage and dysfunction in the parasitized organs (mainly the liver) [13, 14]. Clinically, patients with CE are in the late stage of echinococcosis when they present to the hospital. The cyst is frequently misdiagnosed as a tumour [15]. Therefore, determination of a positive and effective approach to discriminate patients with CE from healthy individuals is important for the early diagnosis and treatment of echinococcosis, which can significantly improve the survival rate of patients.

Metabolomics, a new high-throughput sequencing technology introduced in recent years, is an important branch of omics and has great potential in drug toxicity or safety evaluation [16]. Metabolomics reflects many factors, such as gene changes, nutritional status, pathogenesis, natural environmental changes, drug treatment, physiological response, and pathological characteristics [17–21]. It is a dynamic observation of the occurrence and development of diseases and the transformation process [22]. In metabolomic studies, small molecule metabolites are used as the research object. It can be used to study drug metabolism, find differential metabolites, and explore the target and mechanism of action with the corresponding statistical analysis software using highly sensitive instruments (gas chromatography–mass spectrometry (GC–MS), nuclear magnetic resonance (NMR), and liquid chromatography–mass spectrometry (LC–MS/MS) [23–25]. Metabolomics has been widely used in a variety of hepatopathies to identify potential early biomarkers and metabolic pathways [26], which is a feasible measure for illustrating host–parasite interactions [27–29]. The metabolic state and biochemical activity of cells or tissues can be directly reflected by metabolites such as amino acids, lipids, or sugars [30, 31]. Metabolic profiling approaches have been widely used in various studies on diseases caused by flatworms such as *Fasciola hepatica* and roundworms such as *Onchocerca volvulus* [32–35]. Nevertheless, reports on the application of the LC–MS/MS-based metabolomics approach in echinococcosis are limited [36]. In this study, we used a LC–MS/MS system-based metabolomics approach and multivariate statistical analyses to investigate the molecular mechanism of CE and to provide a potential valuable reference for the diagnosis of CE.

## Methods

### Establishment of hydatidosis model

Twenty 8-week-old female BALB/c mice weighing about 18–22 g were randomly divided into two groups: normal control group (*n* = 10) and *E. granulosus* infection group (*n* = 10). The mice were intraperitoneally injected with protoscoleces diluted with 2 mL phosphate-buffered saline (PBS) (containing 2000 protoscoleces) to develop the *E. granulosus* infection model. The normal control group was intraperitoneally injected with the same 2 mL PBS. The liver and faeces of both groups were obtained by dissection after 6 weeks of feeding. The mice models infected with *E. granulosus* were confirmed through routine pathological section staining.

The animal experiments were implemented in strict accordance with the guidelines for laboratory animal management, with the approval of the Ningxia Medical University animal ethics committee, and in accordance with the National Institutes of Health guidelines for the care and use of laboratory animals.

### Sample preparation

First, 25 mg of hepatic and faecal samples were weighed and transferred to a new clean EP tube. Then, 500 μL extract solution (acetonitrile: methanol: water = 2:2:1) with isotopically labelled internal standard mixture was added to the samples. After 30 s of gentle vortexing, the samples were homogenized at 35 Hz for 4 min followed by ultrasonication in an ice-water bath for 5 min. The homogenization and ultrasonication cycle was carried out three times. Then, the samples were incubated at −40 °C for 1 h and centrifuged at 4 °C for 15 min at 12,000 rpm. The resulting supernatant was transferred to a fresh and clean glass bottle for analysis. A quality control sample was prepared by mixing the supernatants of all samples of liver or faeces aliquoted in equal amounts.

### LC–MS/MS analysis

LC–MS/MS analyses were performed using a high-performance liquid chromatography (HPLC) system (Vanquish, Thermo Fisher Scientific, Waltham, MA, USA and Bruker BioSpin, Karlsruhe, Germany). The injection volume of the liver and faeces was 3 μL.

The LC–MS/MS spectra were collected by a Q Exactive HF-X Orbitrap (QE HFX) mass spectrometer in the control of the acquisition software (Xcalibur, Thermo Fisher Scientific). The acquisition software continuously evaluates the full scan MS spectrum in this mode. The conditions of electrospray ionization were set as follows: auxiliary gas flow rate 10 Arb, capillary temperature 320 °C, sheath gas flow rate 50 Arb, full MS resolution 60,000, collision energy 10/30/60 in NCE mode, LC–MS/MS resolution 7500, and spray voltage 3.5 kV (positive) or −3.2 kV (negative).

### Metabolomics statistical analysis

The original data were converted to mzXML format using ProteoWizard (ProteoWizard, Palo Alto, CA, USA), and processed by an in-house program which was based on XCMS and was developed using R (R Foundation for Statistical Computing, Vienna, Austria). Metabolites were annotated using an in-house MS2 database (BiotreeDB). The cutoff value for annotation was set to 0.3.

### OPLS-DA analysis

The data were log-transformed and UV-formatted using SIMCA software (V16.0.2, Sartorius Stedim Data Analytics AB, Umea, Sweden), and the first principal component was analysed by OPLS-DA modeling. The quality of the model was tested using sevenfold cross-validation.

### Statistical analysis

SPSS software (version 20.0 for Windows; IBM Corporation, Armonk, NY, USA) was used to analyse the data, which have been expressed as mean ± SD. In all cases, *p* < 0.05 was considered to be significant.

## Results

### Multivariate statistical analysis

**Fig. 1:**
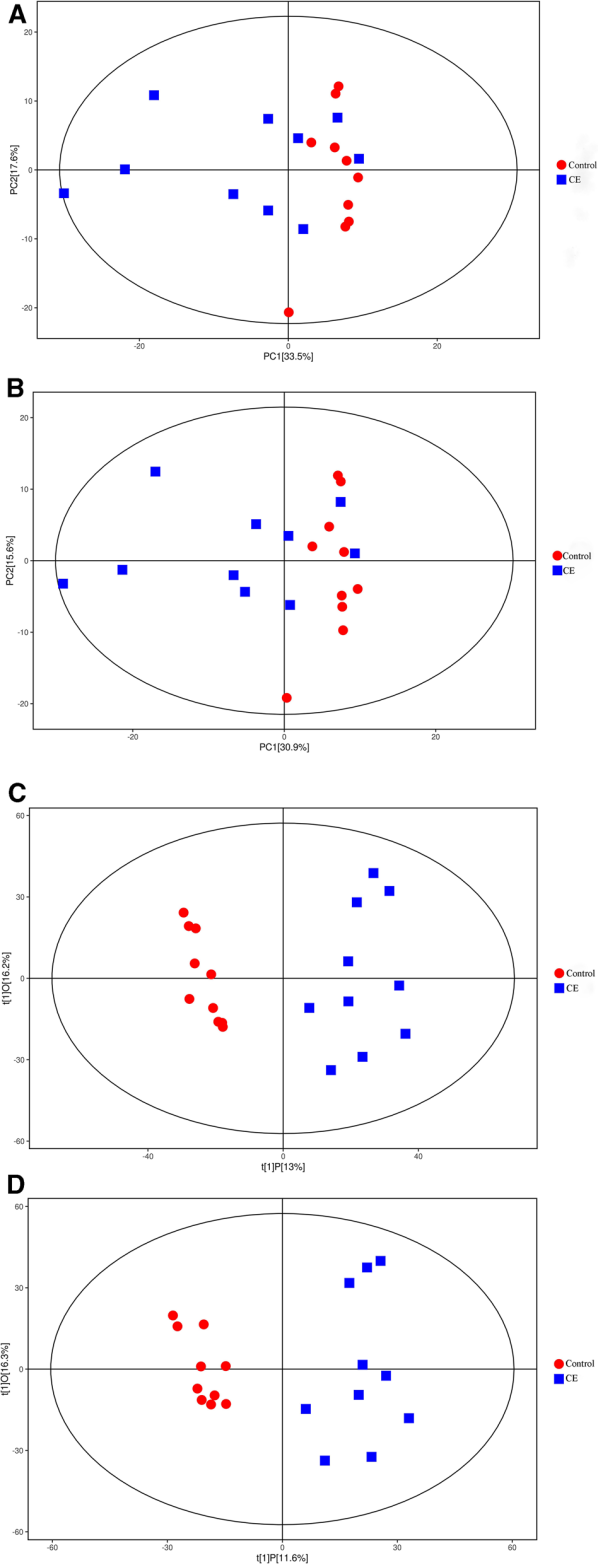
PCA (**A**, **B**), OPLS-DA (**C**, **D**) score plots and OPLS-DA permutation plots (**E**, **F**) in the liver. **A**, **B** PCA score plots, the abscissa PC1 and the ordinate PC2 represent the scores of the principal components ranking the first and the second, respectively, and different shapes of the scattered points represent the different groups of the samples. **C**, **D** OPLS-DA score plots, the ordinate t[1]O represents the orthogonal principal component score, the abscissa t[1]P represents the predicted principal component score of the first principal component, and different shapes of the scattered points represent the different groups of the samples. **E**, **F** OPLS-DA permutation plots. The abscissa correlation coefficient represents relevance. The Q^2^ and R^2^Y values reflect the model predictability and the fraction of explained variance, respectively

To determine the metabolic changes in the liver and faeces due to echinococcosis initiated following *E. granulosus* infection, principal component analysis (PCA) was conducted for two groups of samples. The metabolites of the liver were found to be in moderate groupings between the infection and normal control groups under positive ion mode (POS) and negative ion mode (NEG) according to PCA (Fig. 1A, B). The interpretation rates of the first principal component (PC1) and second principal component (PC2) under POS were 33.5% and 17.6%, respectively, while under NEG, the respective interpretation rates were 30.9% and 15.6%. To better highlight the metabolic changes between the two groups, orthogonal projections to latent structures–discriminant analysis (OPLS-DA) was performed. The difference between the two groups was evident in the OPLS-DA score plots (Fig. 1C, D). A similar tendency was observed in the metabolic changes in faeces (Additional file 1: Fig. S1A–D). The group separation revealed that hydatid disease could cause notable metabolic changes in the liver and faeces of mice. All the samples were within the 95% confidence interval. In addition, the OPLS-DA models were supposed to explain the predictive powers Q^2^ and variations R^2^Y with permutation tests (Fig. 1E, F, Additional file 1: Fig. S1E, F).

### Screening of differential metabolites

**Fig. 2:**
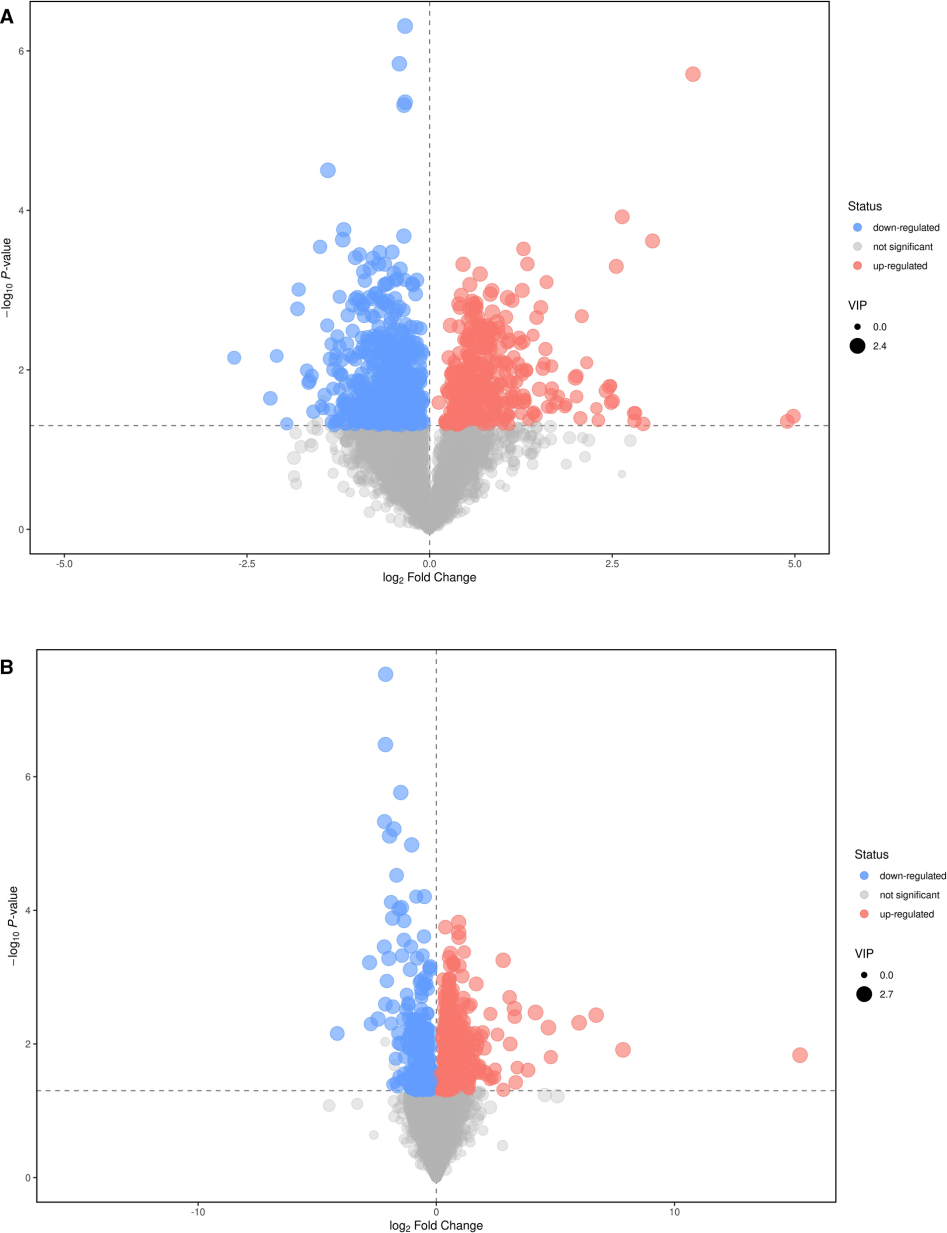
Volcano plots in the liver (**A**, **B**). **A**, **B** Volcano plots. Each dot in the volcano map represents a metabolite, the abscissa shows the fold change value (take the logarithm of cardinal number 2), the ordinate represents the *p* value of Student's *t* test (take the negative number of base logarithm of 10), and the size of the scatter represents the VIP value of the OPLS-DA model: the larger the scatter, the greater the VIP value

To distinguish metabolic markers between the infection and control groups, the data are presented in the form of volcano plots. The results of the metabolic changes were determined based on variable importance in the projection (VIP) > 1 and *p* < 0.05. The metabolites with enormous changes have been represented with blue or red dots and larger circle shapes, located in the upper left or right corner of the volcano plots. From the metabolites of the liver, 248 and 131 distinct metabolic molecules were screened under POS and NEG (Fig. 2A, B). In the faeces, 201 and 58 various metabolites were screened under POS and NEG (Additional file 2: Fig. S2A, B). The top 15 metabolites with the most significantly different metabolism under POS and NEG in the liver and faeces are shown in Tables 1 and 2 and Additional file 3: Tables S1 and S2. In the livers from the infection group, representative substances such as bile acid (deoxyviolaceinic acid), glycerides (2-*O*-(alpha-*d*-mannosyl)-*d*-glycerate), amino acids (glutaminylhistidine), and nucleotides (cytidine 2',3'-cyclic phosphate) were significantly upregulated, while nucleotides (inosinic acid and 5-fluorodeoxyuridine monophosphate) were markedly downregulated under POS and in NEG. Furthermore, in the faeces of the mice with echinococcosis, metabolites such as nucleotides (dTMP), alkaloids (piperine), amino acids (*d*-pantethine, *d*-aspartic acid, and gamma-glutamylleucine), and amides (*N*-acetylhistamine) were upregulated, while nucleosides (5′-methylthioadenosine), amino acids (3-methylcrotonylglycine, selenocystemic acid), organic acids (hexadecanedioic acid, dimethylmalonic acid, and ascorbic acid), and alkaloids (cytokinin b) were downregulated under POS and NEG (Tables 1, 2 and Additional file 3: Tables S1 and S2). The results suggest that the metabolism of nucleotides, alkaloids, amino acids, amides, and organic acids in mice is closely interrelated with *E. granulosus* infection.

**Table 1:** Key differential metabolites of echinococcosis of the liver in POS

Number	Different metabolites	rt	mz	*P* value	Fold change	Variation trend
1	DG(13:0/0:0/a-25:0)	473.96	654.1	0.04433	29.7814283	↑
2	Deoxyviolaceinic acid	52.113	358.37	0.00024	8.29206077	↑
3	Cytidine 2',3'-cyclic phosphate	344.54	306.05	0.04765	7.59016312	↑
4	6-Hydroxyprotopine	129.38	370.37	0.01581	5.57671424	↑
5	(-)-Epiafzelechin 3-gallate	491.7	427.11	0.0162	5.50331511	↑
6	CGP 28–392	132.37	366.34	0.02168	4.03262168	↑
7	2-(2,4-dihydroxyphenyl)-5-hydroxy-8-(hydroxymethyl)-8-methyl-4H,8H-pyrano[3,2-g]chromen-4-one	130.6	369.36	0.01196	4.02562678	↑
8	2-Phospho-4-(cytidine 5'-diphospho)-2-C-methyl-D-erythritol	413.81	602.31	0.0083	2.96650399	↑
9	Bicuculline	130.42	368.35	0.00962	2.92640942	↑
10	Methylselenopyruvate	457.16	182.06	0.01751	2.83446931	↑
11	2-O-(alpha-D-Mannosyl)-D-glycerate	193.08	269.23	0.01133	2.40617468	↑
12	Glutaminylhistidine	389.01	284.13	0.01026	2.39624637	↑
13	4-Bromocatechol	190.44	190.01	0.03537	2.37914101	↑
14	D-Erythrose 4-phosphate	146.56	201.09	0.00465	2.36957829	↑
15	PC(DiMe(11,3)/DiMe(11,3))	448.5	868.17	0.02601	2.31441094	↑
16	Asulam	327.76	372.83	0.00573	0.22063809	↓
17	PE(20:3(5Z,8Z,11Z)/24:1(15Z))	327.76	568.78	0.00719	0.28871571	↓
18	2,2-Bis(4-hydroxyphenyl)hexafluoropropane	327.75	960.69	0.02199	0.35378049	↓
19	Glucoconringiin	327.75	725.8	0.02367	0.36436639	↓
20	Aminosalicylate sodium anhydrous	630.94	279.09	0.00591	0.40899599	↓
21	2'-O-Methyladenosine	327.75	335.9	0.01821	0.42089842	↓
22	Chlorfenson	327.76	668.76	0.00292	0.4254822	↓
23	Urate radical	327.74	764.74	0.00112	0.43010083	↓
24	Inosinic acid	175.43	120.07	0.01828	0.43609542	↓
25	2-Oxoadipate	239.73	240.1	0.0474	0.43874004	↓
26	Perfluorooctane sulfonamide	162.66	796.58	0.0371	0.44433904	↓
27	3'-Keto-3'-deoxy-ATP	402.81	161.14	0.01662	0.46859336	↓
28	l-trans-4-Methyl-2-pyrrolidinecarboxylic acid	327.72	962.69	0.00548	0.47930656	↓
29	(2S,4R,5S)-Muscarine	170.55	398.76	0.02404	0.48133876	↓
30	Cyanidin 3-(3'',6''-dimalonylglucoside)	439.59	178.04	0.02354	0.4816437	↓

"↑" and "↓" mean that the compound is upregulated and downregulated

**Table 2:** Key differential metabolites of echinococcosis of the liver in NEG

Number	Different metabolites	rt	mz	*P* value	Fold Change	Variation trend
1	2-Ethyl-2-Hydroxybutyric acid	134.31	131.07	0.01472	39,455.9465	↑
2	MC-7181	171	361.13	0.00569	26.0838692	↑
3	2-Hydroxyvaleric acid	159.08	117.05	0.00338	17.9190231	↑
4	Cytidine 2',3'-cyclic phosphate	344.45	304.03	0.03763	10.0652201	↑
5	DG(11D3/13M5/0:0)	491.15	744.15	0.002	8.42980787	↑
6	Sodium citrate	159.1	295.1	0.00056	6.99280125	↑
7	3-[(2,6-Dichlorobenzylidene)amino]-6H-dibenzo[b,d]pyran-6-one	227.4	369.23	0.02382	5.54178921	↑
8	Carboxytolbutamide	302.59	299.07	0.00355	4.82213854	↑
9	Methylselenopyruvate	491.45	182.06	0.01041	3.32312262	↑
10	3-Methyl-2-oxovaleric acid	168.37	129.06	0.01252	3.20319072	↑
11	Mandipropamid	475.68	412.88	0.01769	2.95950439	↑
12	Pamidrote	302.62	236.08	0.01395	2.86378385	↑
13	Soyacerebroside I	361.23	715.04	0.03372	2.65108725	↑
14	Desflurane	491.6	169.05	0.01274	2.51125096	↑
15	2,2-Bis(bromomethyl)propane-1,3-diol	540.32	262.95	0.00742	2.27605124	↑
16	Diclofop	218.27	328.16	4.7E-06	0.22147421	↓
17	Vinclozolin	31.239	287.11	3.3E-07	0.22739657	↓
18	Disul	30.407	288.12	6.1E-06	0.28955467	↓
19	2,5-Dichloro-4-oxohex-2-enedioate	26.775	228.01	0.00997	0.35460978	↓
20	Histamine Phosphate	28.593	308.14	1.7E-06	0.35537393	↓
21	Pyronaridine	495.59	519.05	0.01564	0.39746798	↓
22	Bis(4-nitrophenyl)phosphate	27.675	341.2	0.00305	0.41584266	↓
23	5-Fluorodeoxyuridine monophosphate	26.426	327.18	0.00182	0.42170291	↓
24	1-(5-Phospho-D-ribosyl)-5-amino-4-imidazolecarboxylate	27.688	340.2	0.00261	0.44501339	↓
25	3,4,8,9,10-Pentahydroxy-6-oxo-6H-benzo[c]chromene-1-carboxylic acid	24.936	321.21	0.00769	0.44670044	↓
26	Inosinic acid	466.43	347.04	0.01181	0.44738538	↓
27	DG(22:4(7Z,10Z,13Z,16Z)/24:1(15Z)/0:0)	509.7	756.22	0.03233	0.4703637	↓
28	5-Phosphoribosyl-4-carboxy-5-aminoimidazole	28.575	337.18	0.0055	0.47052869	↓
29	Tolclofos-methyl	28.555	302.14	0.00035	0.47796071	↓
30	Maduropeptin chromophore	417.12	780.24	0.03851	0.48066398	↓

"↑" and "↓" mean that the compound is up-regulated and down-regulated

### Hierarchical clustering analysis of different metabolites

**Fig. 3:**
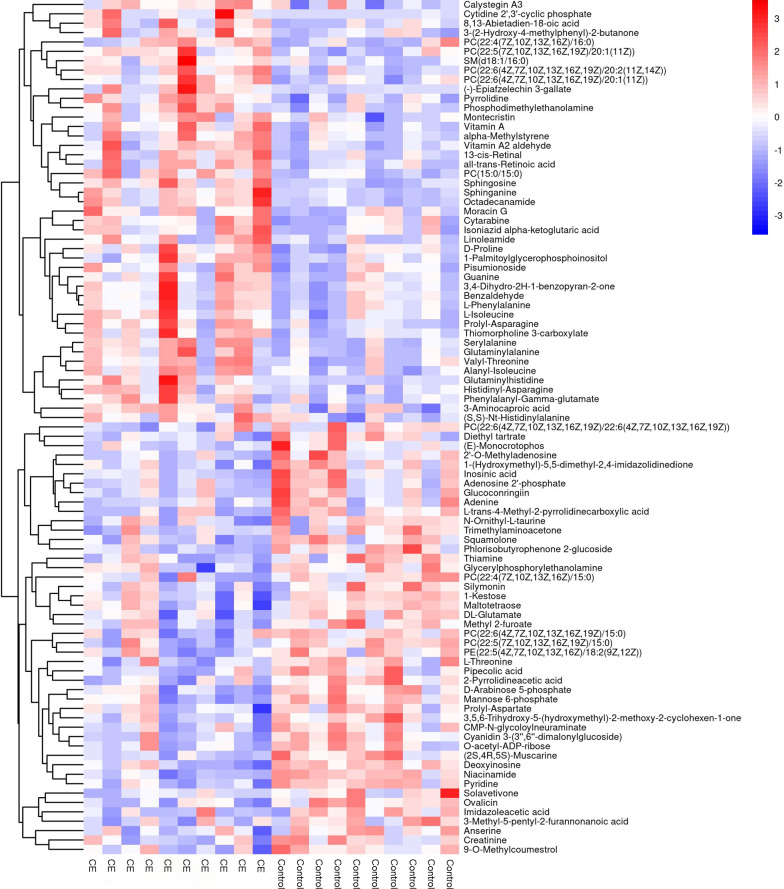

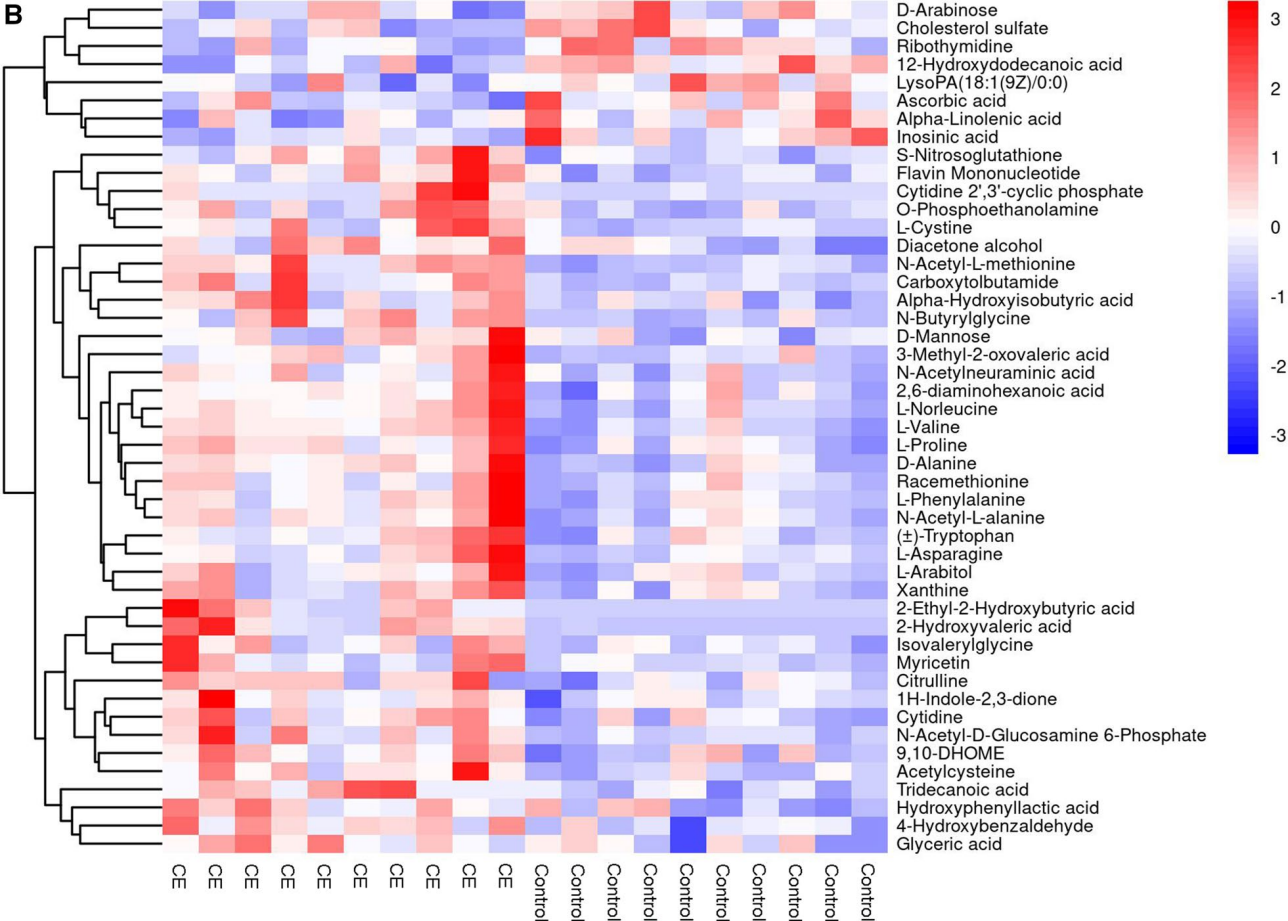
Heat map in the liver. **A**, **B** The abscissa indicates different experimental groups, the ordinate means different metabolites compared with the normal control group, and the square coloured blocks at different spaces represent the relative expression of metabolites at corresponding positions

For every sample, we calculated the Euclidean distance matrix to determine the quantitative value of the differential metabolites. The differential metabolites of the liver were clustered through the complete linkage method and displayed in the thermodynamic diagram (Fig. 3A, B). Through further evaluation, we selected 91 and 47 differential metabolic molecules under POS and NEG. Liver metabolites mainly included esters, amino acids, carbohydrates, and lipids. Compared with the normal control group, the expression of glyceric acid, acetylcysteine, myricetin, l-arabitol, l-asparagine, tryptophan, l-phenylalaine, racemethionine, d-alanine, d-proline, l-valine, and d-mannose were all increased in the infection group. Conversely, in the infection groups the levels of cholesterol sulfate, ribothymidine, ascorbic acid, alpha-linolenic acid, inosinic acid, adenine, thiamine, maltotetraose, creatinine, and imidazoleacetic acid were evidently decreased. Additionally, the levels of other metabolites were changed to various degrees. Faecal metabolic molecules were analysed similarly; 107 and 23 differential molecules were selected under POS and NEG (Additional file 4: Fig. S3A, B). The expression of metabolic molecules such as medicagenic acid, anandamide, piperine, N-carbamoyl putrescine, isoleucyl phenylalanine, d-aspartic acid, cytidine, and l-valine were sharply increased in the infection group. Besides, metabolic molecules such as trimethylaminoacetone, histidine, ophthalmic acid, hexadecanedioic acid, methylthioadenosine, traumatic acid, and phenylacetic acid showed a downward trend in the infection group. Coincidentally, l-valine was upregulated in the liver and faeces in the infection group. The heat map (Fig. 3A, B) shows that the different metabolites had good classification results.

### Correlation analysis of different metabolites

**Fig. 4:**
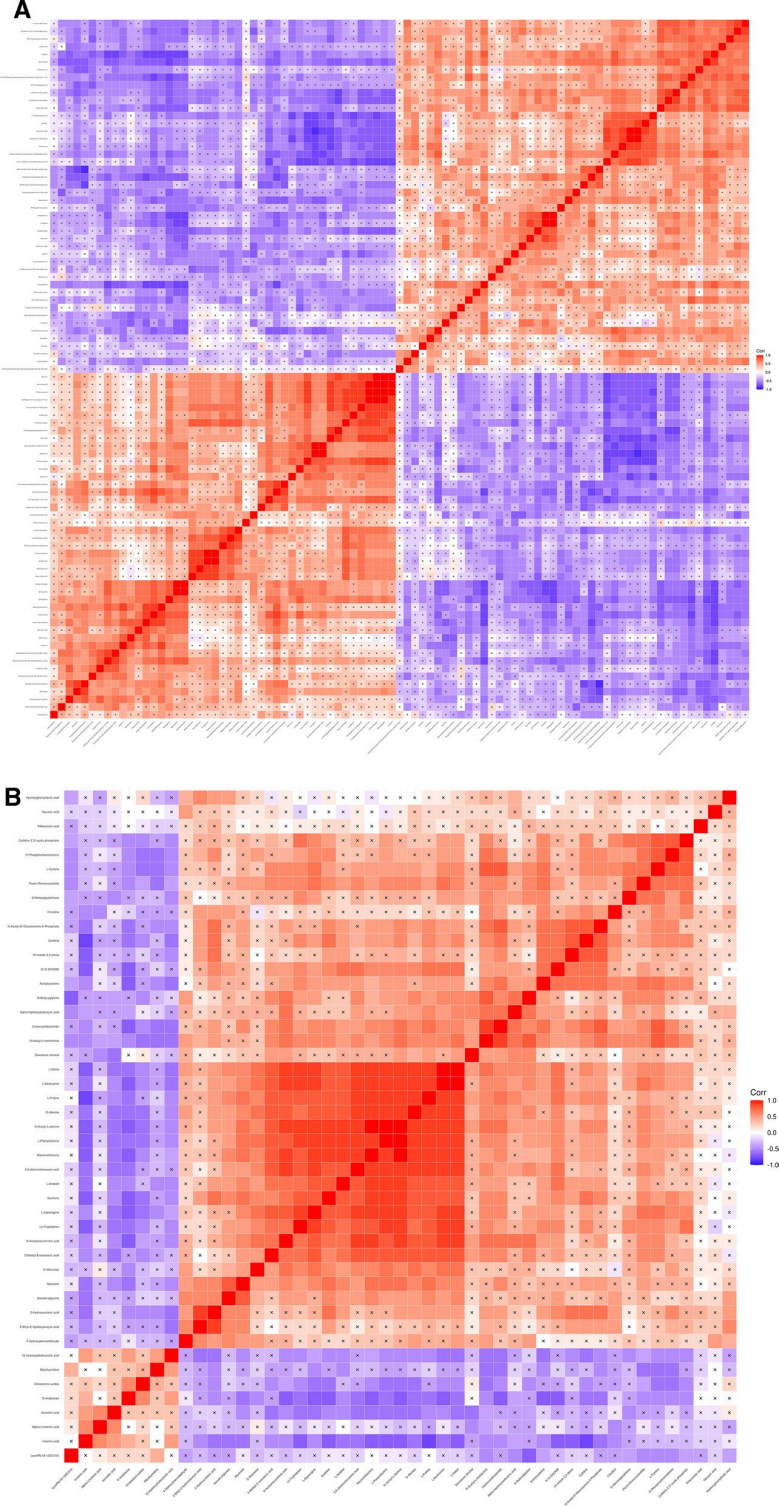
Heat map of correlation analysis by group in the liver. **A**, **B** The abscissa and ordinate represent the different metabolites of the group comparison. The square coloured blocks in different positions represent the correlation coefficient between the two metabolites at corresponding positions. Red shows positive correlation, blue shows negative correlation, and the darker the colour, the stronger the correlation. Nonsignificant correlations are marked with a cross

Complex metabolic reactions and their regulation did not occur in isolation after the mice were infected with *E. granulosus*. When different molecules work together, their interaction and regulation eventually lead to systematic changes in metabolomics. We calculated the correlation coefficient of the quantitative values of different metabolites and expressed it as *r*. In this study, 91 and 47 differential metabolic molecules in the liver under POS and NEG were analysed using the Pearson method. There was a clear correlation between amino acid metabolism (Fig. 4A, B, *p* < 0.05). We also found that 107 and 23 differential molecules in the faeces observed under POS and NEG were related (Additional file 5: Fig. S4A, B, *p* < 0.05).

### KEGG annotation of different metabolites

**Fig. 5:**
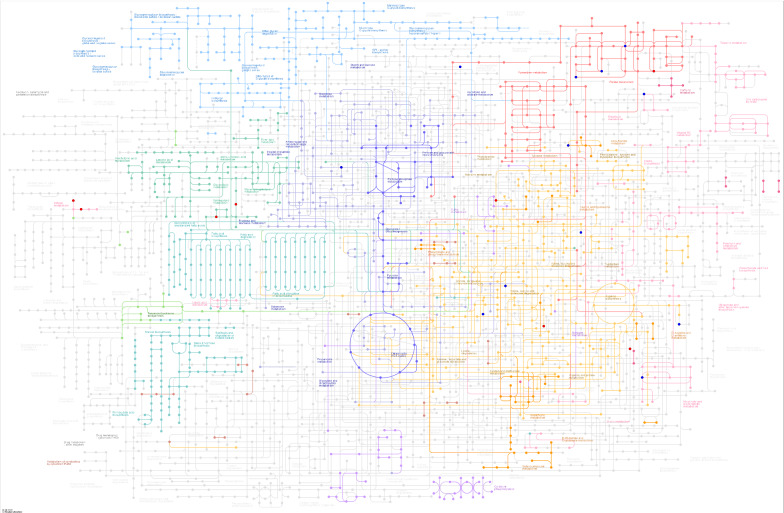

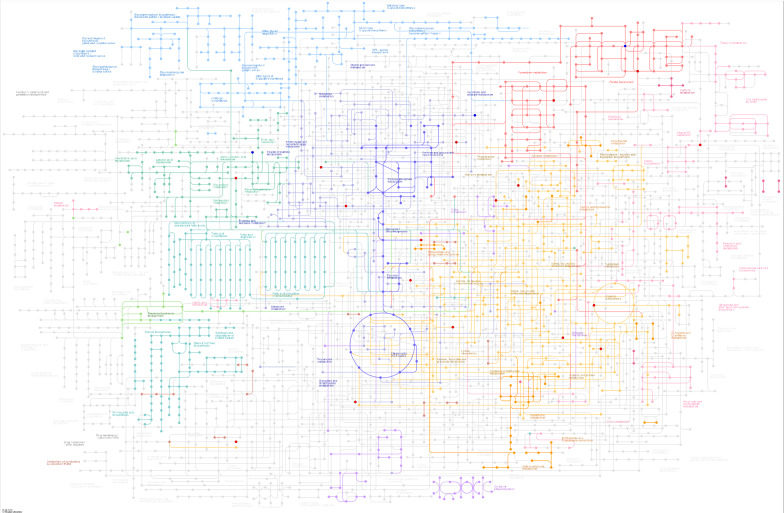
KEGG pathways map in the liver. **A**, **B** KEGG pathways map; the red and blue dots indicate the metabolic pathways involved by the differentially expressed metabolites

From the KEGG pathway map, we found that the differential metabolic molecules between the infection and control groups involved multiple pathways, including energy metabolism, material transport, signal transduction, and cell cycle regulation (Fig. 5A, B). In all, 46 pathways in the liver were involved with the differential metabolic molecules. In the faeces, these differentially metabolized molecules involved 57 pathways (Additional file 6: Fig. S5A, B). For example, in the liver of the infected group, there were four differentially metabolized molecules that were enriched in purine metabolic pathways and three differentially metabolized molecules that were enriched in amino acid metabolic pathways. Similarly, in the faeces of the infected group, three differential metabolic molecules were involved in pyrimidine metabolism, and two differential molecules were involved in arginine and proline metabolism. The more the number of differential metabolic molecules involved in a certain pathway, the more notable is the difference in the metabolic pathway between the infection and control groups.

### Analysis of metabolic pathways of different metabolites

**Fig. 6:**
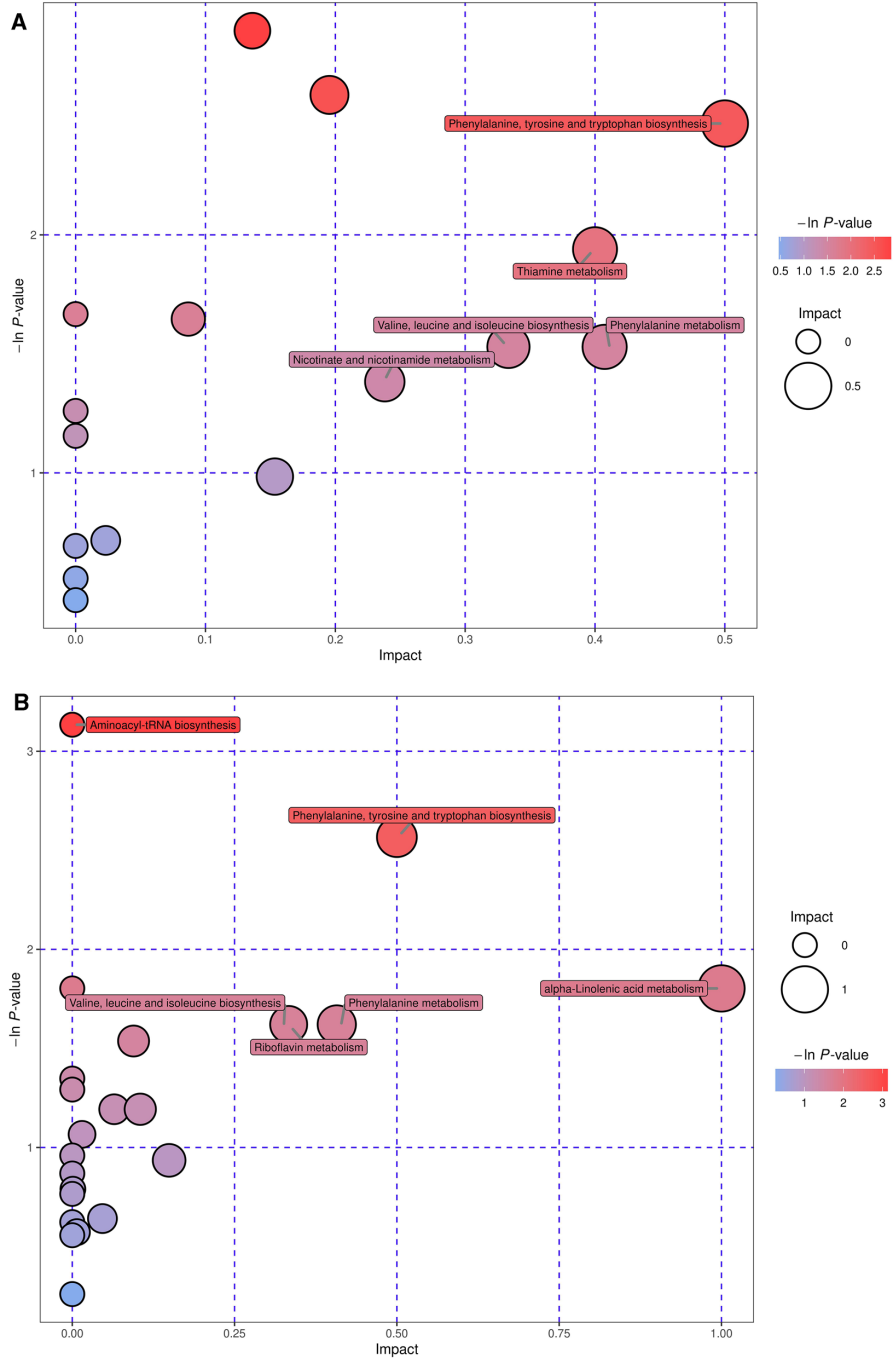
Pathway analysis by group in the liver. **A**, **B** Pathway analysis by group. In the bubble plots, different bubbles represent different metabolic pathways. The abscissa and the size of bubbles indicate the influence factor of the pathway in topological analysis: the larger the size, the greater the influence factors. The ordinate and the colour of the bubble show the *p* value of enrichment analysis (negative natural pair, i.e.—in (P)). The deeper the colour, the smaller the *p* value, and the more significant the enrichment degree

KEGG annotation analysis revealed all the pathways involving the differential metabolites. To determine whether these metabolic pathways were closely related to the experimental conditions, it was necessary to further analyse the metabolic pathways of the differential metabolites. By analysing the synthesis of the pathways (including topological analysis and enrichment analysis), we could further explore the pathways and determine the key pathways with the highest correlation with metabolic differences. The results of the metabolic pathway analysis are presented using bubble charts. In the liver, there were seven metabolic pathways under POS and NEG, three of which were shared. The metabolic pathways of tyrosine and tryptophan biosynthesis, phenylalanine, valine, leucine, and isoleucine biosynthesis and phenylalanine metabolism were notably associated with the occurrence and development of hydatid disease (Fig. 6A, B). However, there were 15 metabolic pathways under POS and NEG, one of which was shared (Additional file 7: Fig. S6A, B), which revealed that pantothenate and CoA biosynthesis is also significantly related to echinococcosis.

## Discussion

In this study, we analysed the metabolic footprint and significant changes in metabolism, which revealed the substantial changes in the mice metabolome caused by CE using the multivariate statistical analysis of liver and faeces. In all, 138 metabolic molecules were selected to discriminate CE mice from healthy mice. Seven metabolic pathways appeared under POS and NEG that may be related to echinococcosis. The three common metabolic pathways were tyrosine and tryptophan biosynthesis; phenylalanine, valine, leucine, and isoleucine biosynthesis; and phenylalanine metabolism, which indicated that amino acid metabolism is strongly associated with the occurrence and development of hydatid disease. These metabolic differences may provide novel insight into the biological mechanisms that occur during CE infection. Interestingly, 130 metabolic molecules in faeces were considered to distinguish between the infection and control groups. These molecules were enriched in 15 pathways, one of which is in common under POS and NEG. The pantothenate and CoA biosynthesis pathway indicated that the metabolism of fatty acid and pyruvate in mice with echinococcosis would be disturbed.

The main organ involved in echinococcosis is the liver. With cyst parasitization and growth, it continuously obtains nutrition from the liver. When it grows large enough, it induces change in the structure and function of the liver [1, 37]. The results revealed that CE can lead to significant changes in amino acid metabolism. The circulation level of amino acids indicates the equilibrium between muscle and liver metabolism [38].

These results were confirmed by the increased phenylalanine and tyrosine levels in the infection group. Tyrosine is the primary product of phenylalanine. It has been reported that the concentrations of these two metabolites are the same. The conversion of phenylalanine to tyrosine occurs uniquely in the liver [39, 40]. The catabolism of amino acids occurs mainly in the liver [41]. Liver injury can lead to changes in amino acid metabolism, mainly manifested as a decrease in free branched-chain amino acids and an increase in free amino acids (phenylalanine, tryptophan, and tyrosine) [42]. In contrast with the normal mice, the molecular metabolites of the liver and faeces were related in the infected mice. Among the first 15 most differentially metabolized molecules, tolclofos methyl was increased in the liver and reduced in the faeces and chlorfenson was downregulated in the liver and upregulated in the faeces, while aminosalicylate sodium anhydrous and Disul were both decreased in the liver and faeces. In conclusion, the reason why amino acids (phenylalanine, tyrosine, tryptophan, valine, leucine, and isoleucine) were increased in the infection group may be related to liver dysfunction. We noticed that purine metabolism and alpha-linolenic acid metabolism were abnormal in CE mice, which suggests that other pathways may participate in the metabolism after hydatid infection as well.

The limitations of this study were as follows. First, additional samples are needed to verify the current results and further improve the reliability and accuracy of the procedure. Secondly, the comprehensive application of LC–MS/MS with GC–MS or NMR will expand the coverage of metabolomics. Moreover, a variety of omics technologies (such as genomics and proteomics) can be cross-validated and may better support the experimental results. Finally, no effective method was determined to distinguish echinococcosis from other types of liver disease in this study. Therefore, imaging examination remains the most commonly used method for CE. If the method is developed, it is essential to preventing clinical misdiagnosis because echinococcosis is similar to other hepatic diseases. The feasibility of using the Fischer ratio and unique metabolic characteristics of echinococcosis to identify different liver diseases warrants further study. Certainly, finding a way to reduce the cost of LC–MS/MS could help to accelerate its use in clinical application.

The LC–MS/MS method is suitable for metabolomics analysis of hydatid disease due to its repeatability, multiple metabolite coverage in one measurement, and short detection duration. The systematic research of multiple metabolites of small molecules is helpful in revealing the overall metabolic changes induced by *E. granulosus*. The early diagnosis of echinococcosis may be feasible by combining both imaging techniques and metabolomics. For example, chemical-exchange-weighted magnetic resonance imaging techniques are used to yield maps weighted by the metabolites of interest [43–45]. These maps are obtained by exchanging with water and characterized by significant enhancement of the signal and the observation of small metabolic changes that cannot be observed with other imaging methods. In short, LC–MS/MS-based metabolomics with specificity can be combined to enhance the analysis and better explain the data.

## Conclusion

In our study, LC–MS/MS-based metabolomics was used to investigate metabolic diversification of CE. It was confirmed that amino acid metabolism (phenylalanine, tyrosine, tryptophan, valine, leucine, and isoleucine) and pantothenate and CoA biosynthesis changed significantly following hydatid infection. The specific metabolic changes determined in this study may provide a new understanding of the molecular mechanism of CE as well as some meaningful clues regarding the early diagnosis and therapeutic intervention in CE.

## Supplementary Information

**Additional file 1: Fig. S1.** PCA (A and B), OPLS-DA (C and D) score plots and OPLS-DA permutation plots (E and F) in faeces. A and B. PCA score plots, the abscissa PC1, and the ordinate PC2 represent the scores of the principal components ranking the first and the second, respectively, and different shapes of the scattered points represent the different groups of the samples. C and D. OPLS-DA score plots, the ordinate t[1]O represents the orthogonal principal component score, the abscissa t[1]P represents the predicted principal component score of the first principal component, and different shapes of the scattered points represent the different groups of the samples. E and F. OPLS-DA permutation plots. The abscissa correlation coefficient represents relevance. The Q^2^ and R^2^Y values reflect the model predictability and the fraction of explained variance, respectively.

**Additional file 2: Fig. S2.** Volcano plots in faeces (A and B). A and B. Volcano plots, each dot in the volcano map represents a metabolite, the abscissa shows the fold change value (take the logarithm of cardinal number 2), the ordinate represents the *p* value of Student's *t* test (take the negative number of base logarithm of 10), and the size of the scatter represents the VIP value of the OPLS-DA model: the larger the scatter, the greater the VIP value.

**Additional file 3: Table S1.** Key differential metabolites of echinococcosis of faeces in POS. **Table S2**. Key differential metabolites of echinococcosis of faeces in NEG

**Additional file 4: Fig. S3.** Heat map in faeces (A and B). A and B. The abscissa indicates different experimental groups, the ordinate means different metabolites compared with the normal control group, and the square coloured blocks at different spaces represent the relative expression of metabolites at corresponding positions.

**Additional file 5: Fig. S4.** Heat map of correlation analysis for group in faeces (A and B). A and B. The abscissa and ordinate represent the different metabolites of the group comparison. The square coloured blocks in different positions represent the correlation coefficient between the two metabolites at corresponding positions. Red shows positive correlation, blue shows negative correlation, and the darker the colour, the stronger the correlation. At the same time, the nonsignificant correlation was marked with a cross.

**Additional file 6: Fig. S5.** KEGG pathways map in faeces (A and B). A and B. KEGG pathways map. The red and blue dots indicate the metabolic pathways involved by the differentially expressed metabolites.

**Additional file 7: Fig. S6.** Pathway analysis for group in faeces (A and B). A and B. Pathway analysis for group. In the bubble plots, different bubbles represent different metabolic pathway. The abscissa and the size of the bubble indicate the influence factor of the pathway in topological analysis. The larger the size, the greater the influence factors. The ordinate and the colour of the bubble show the *p* value of enrichment analysis (negative natural pair, i.e. - in (P)). The deeper the colour, the smaller the *p* value, and the more significant the enrichment degree.

## Data Availability

The data of the results of this study are included in this paper and its attached files.

## Abbreviations

LC–MS/MS: Liquid chromatography coupled with tandem mass spectroscopy
CE: Cystic echinococcosis
PCA: Principal component analysis
POS: Positive ion mode
NEG: Negative ion mode
IDA: Information-dependent acquisition
OPLS-DA: Orthogonal projections to latent structures–discriminant analysis
