# Supplementary figures and images for: Metabolic profiling of liver and faeces in mice infected with echinococcosis
Source: Parasit Vectors. 2021 Jun 14;14:324. doi: 10.1186/s13071-021-04807-1 (PMC8201681; doi:10.1186/s13071-021-04807-1)

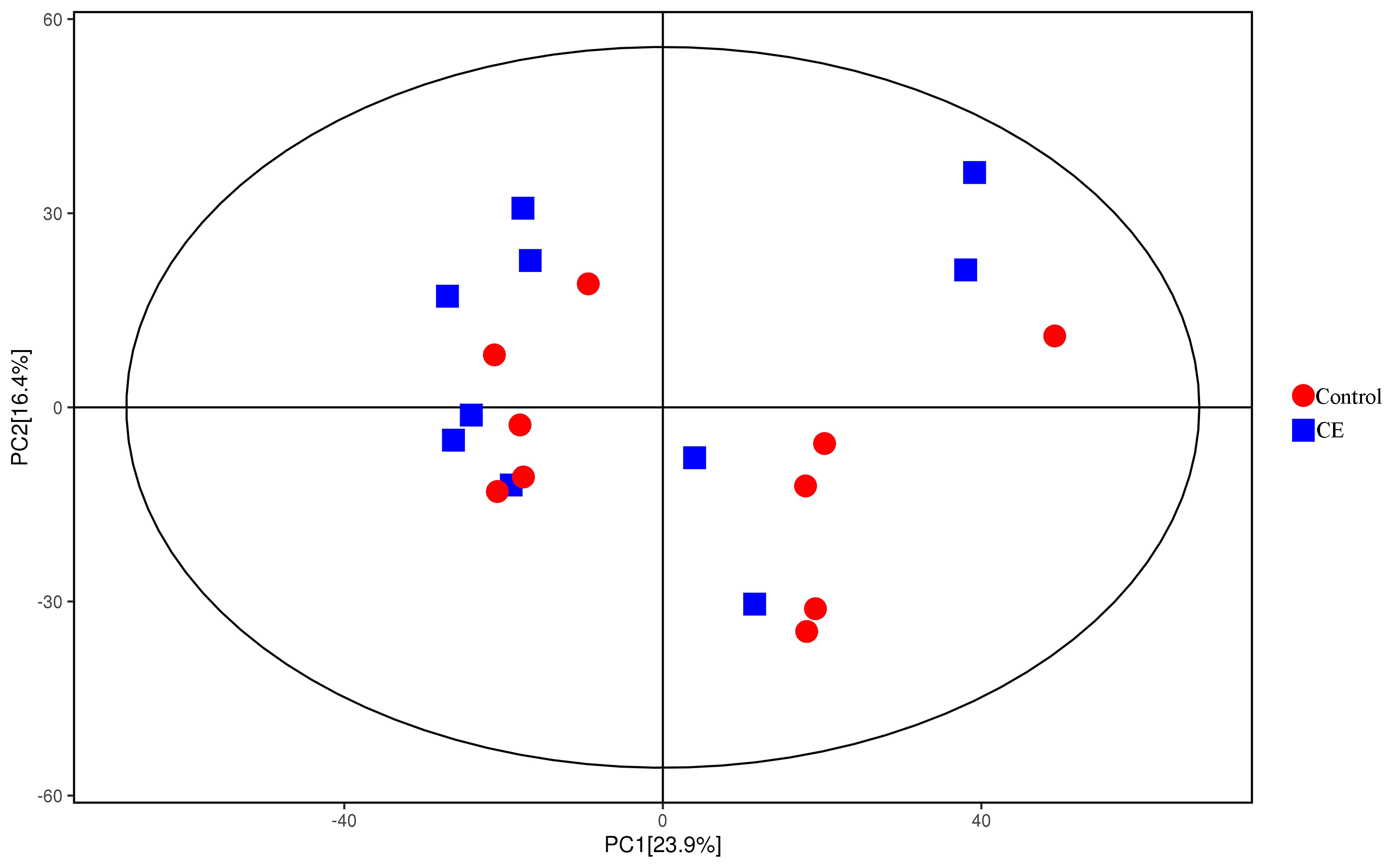

Supplement: Supplementary file 1 — Additional file 1: Fig. S1. PCA (A and B), OPLS-DA (C and D) score plots and OPLS-DA permutation plots (E and F) in faeces. A and B. PCA score plots, the abscissa PC1, and the ordinate PC2 represent the scores of the principal components ranking the first and the second, respectively, and different shapes of the scattered points represent the different groups of the samples. C and D. OPLS-DA score plots, the ordinate t[1]O represents the orthogonal principal component score, the abscissa t[1]P represents the predicted principal component score of the first principal component, and different shapes of the scattered points represent the different groups of the samples. E and F. OPLS-DA permutation plots. The abscissa correlation coefficient represents relevance. The Q2 and R2Y values reflect the model predictability and the fraction of explained variance, respectively. [file 13071_2021_4807_MOESM1_ESM.zip › Sfig1A POS PCA score plot.tif]

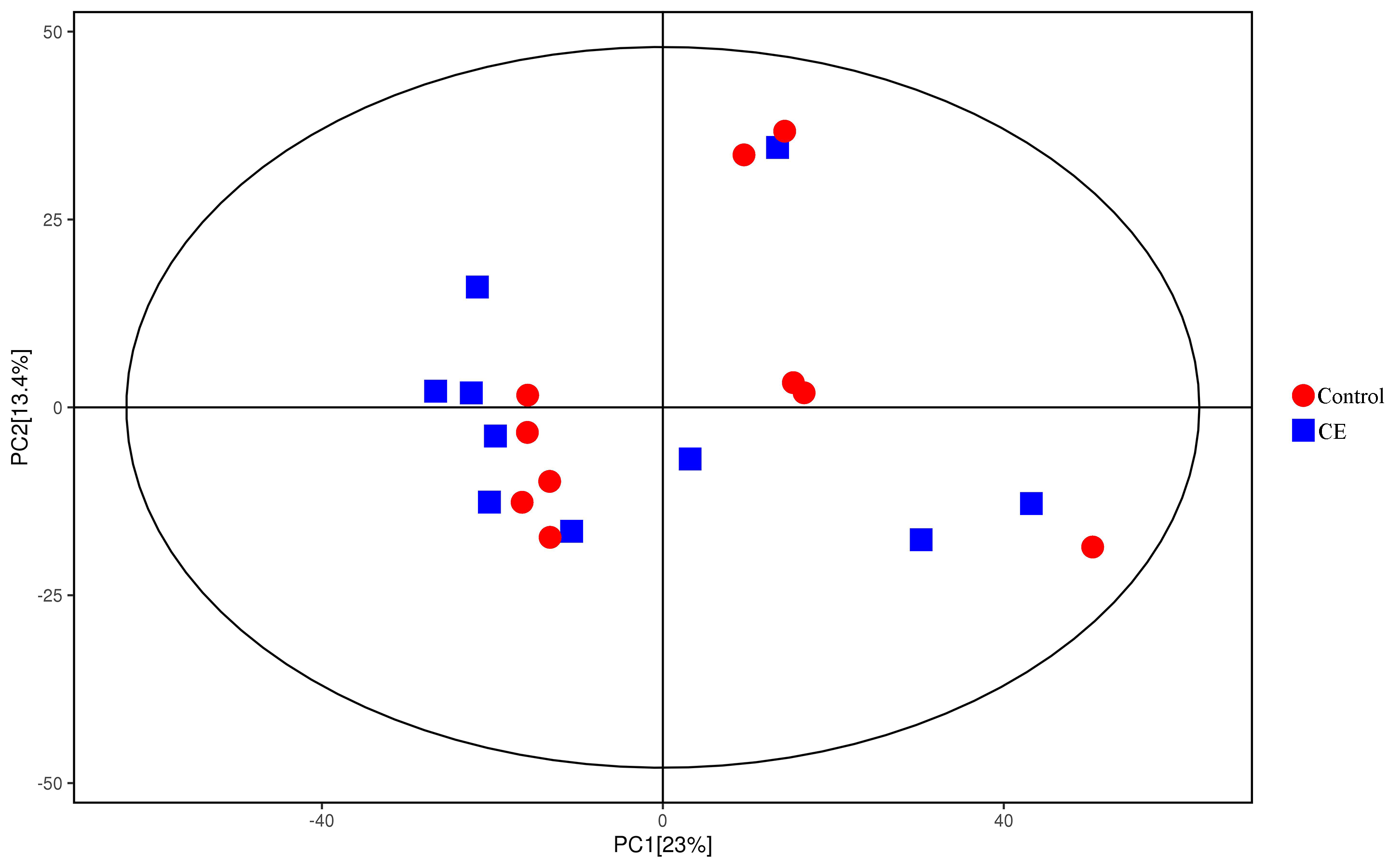

Supplement: Supplementary file 1 — Additional file 1: Fig. S1. PCA (A and B), OPLS-DA (C and D) score plots and OPLS-DA permutation plots (E and F) in faeces. A and B. PCA score plots, the abscissa PC1, and the ordinate PC2 represent the scores of the principal components ranking the first and the second, respectively, and different shapes of the scattered points represent the different groups of the samples. C and D. OPLS-DA score plots, the ordinate t[1]O represents the orthogonal principal component score, the abscissa t[1]P represents the predicted principal component score of the first principal component, and different shapes of the scattered points represent the different groups of the samples. E and F. OPLS-DA permutation plots. The abscissa correlation coefficient represents relevance. The Q2 and R2Y values reflect the model predictability and the fraction of explained variance, respectively. [file 13071_2021_4807_MOESM1_ESM.zip › Sfig1B NEG PCA score plot.tif]

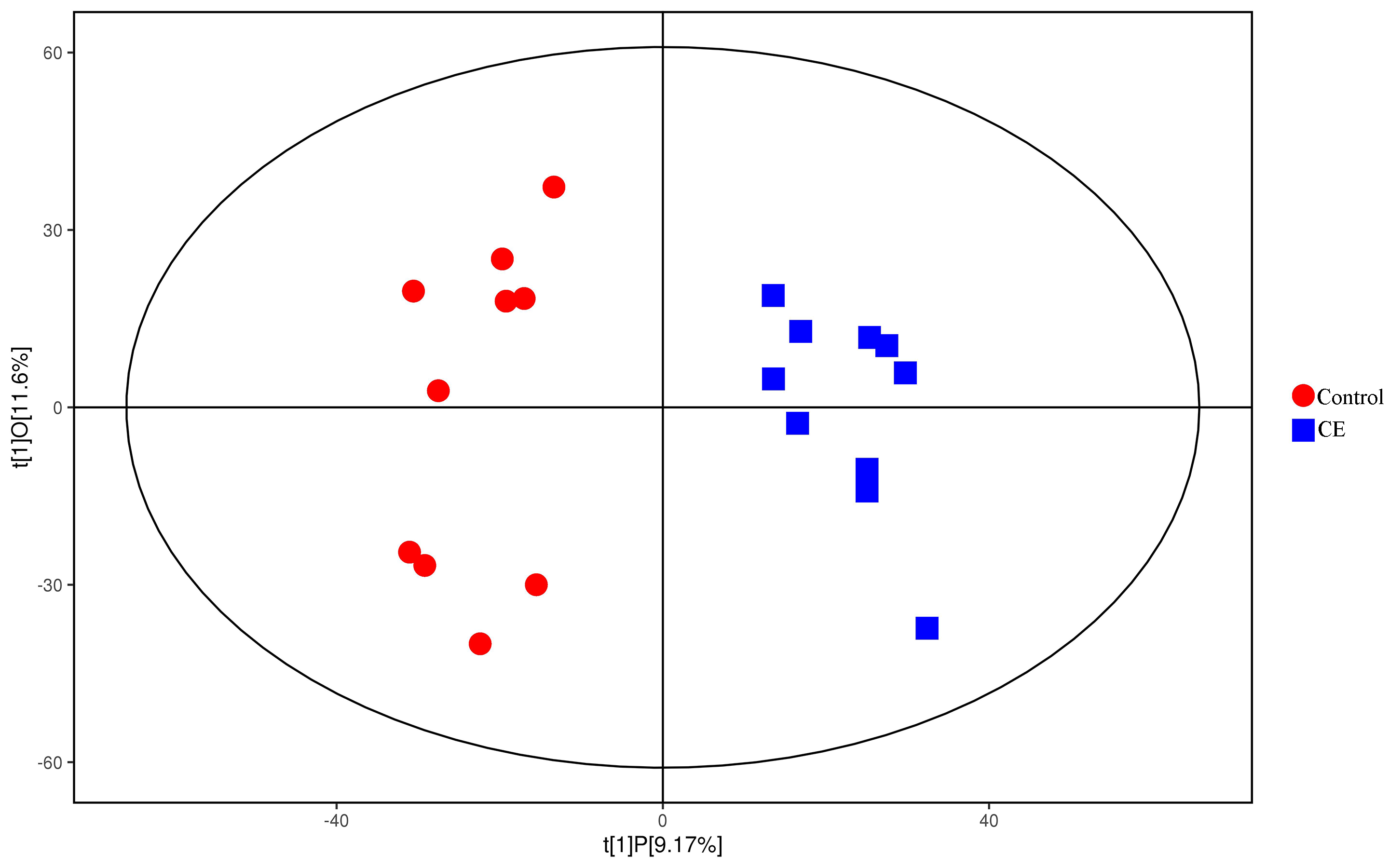

Supplement: Supplementary file 1 — Additional file 1: Fig. S1. PCA (A and B), OPLS-DA (C and D) score plots and OPLS-DA permutation plots (E and F) in faeces. A and B. PCA score plots, the abscissa PC1, and the ordinate PC2 represent the scores of the principal components ranking the first and the second, respectively, and different shapes of the scattered points represent the different groups of the samples. C and D. OPLS-DA score plots, the ordinate t[1]O represents the orthogonal principal component score, the abscissa t[1]P represents the predicted principal component score of the first principal component, and different shapes of the scattered points represent the different groups of the samples. E and F. OPLS-DA permutation plots. The abscissa correlation coefficient represents relevance. The Q2 and R2Y values reflect the model predictability and the fraction of explained variance, respectively. [file 13071_2021_4807_MOESM1_ESM.zip › Sfig1C POS OPLS-DA score plot.tif]

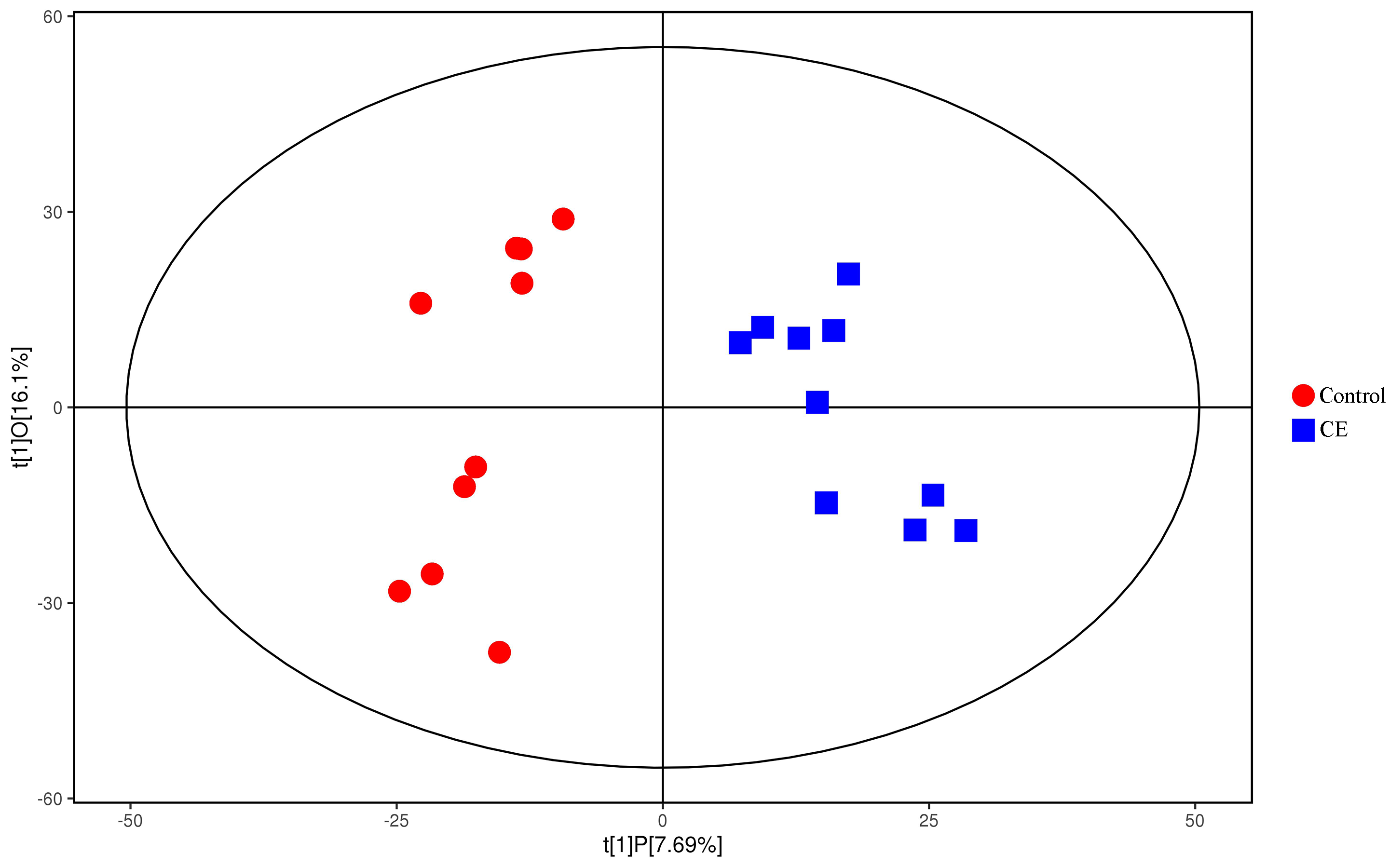

Supplement: Supplementary file 1 — Additional file 1: Fig. S1. PCA (A and B), OPLS-DA (C and D) score plots and OPLS-DA permutation plots (E and F) in faeces. A and B. PCA score plots, the abscissa PC1, and the ordinate PC2 represent the scores of the principal components ranking the first and the second, respectively, and different shapes of the scattered points represent the different groups of the samples. C and D. OPLS-DA score plots, the ordinate t[1]O represents the orthogonal principal component score, the abscissa t[1]P represents the predicted principal component score of the first principal component, and different shapes of the scattered points represent the different groups of the samples. E and F. OPLS-DA permutation plots. The abscissa correlation coefficient represents relevance. The Q2 and R2Y values reflect the model predictability and the fraction of explained variance, respectively. [file 13071_2021_4807_MOESM1_ESM.zip › Sfig1D NEG OPLS-DA score plot.tif]

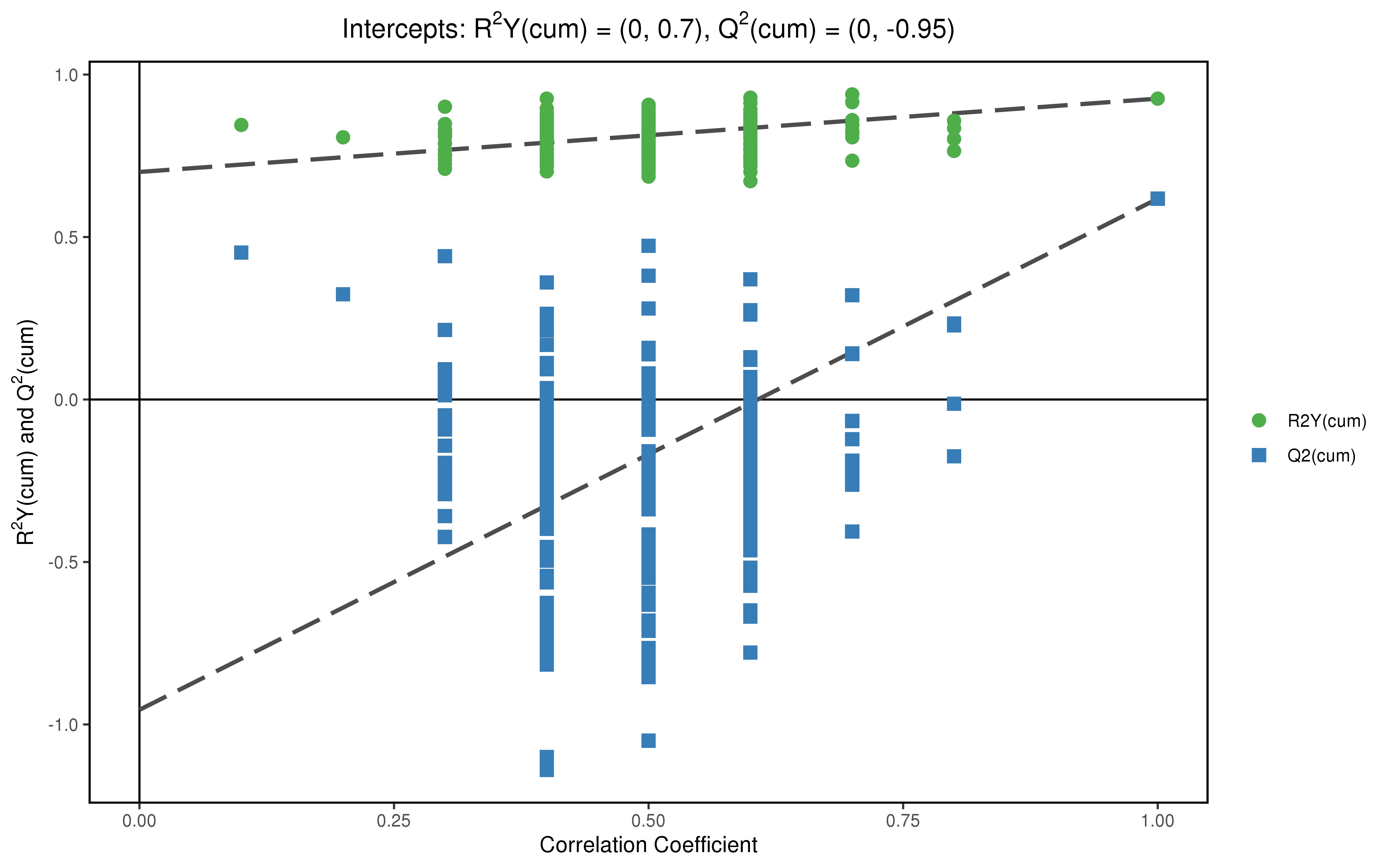

Supplement: Supplementary file 1 — Additional file 1: Fig. S1. PCA (A and B), OPLS-DA (C and D) score plots and OPLS-DA permutation plots (E and F) in faeces. A and B. PCA score plots, the abscissa PC1, and the ordinate PC2 represent the scores of the principal components ranking the first and the second, respectively, and different shapes of the scattered points represent the different groups of the samples. C and D. OPLS-DA score plots, the ordinate t[1]O represents the orthogonal principal component score, the abscissa t[1]P represents the predicted principal component score of the first principal component, and different shapes of the scattered points represent the different groups of the samples. E and F. OPLS-DA permutation plots. The abscissa correlation coefficient represents relevance. The Q2 and R2Y values reflect the model predictability and the fraction of explained variance, respectively. [file 13071_2021_4807_MOESM1_ESM.zip › Sfig1E POS OPLS-DA permutation plot.jpg]

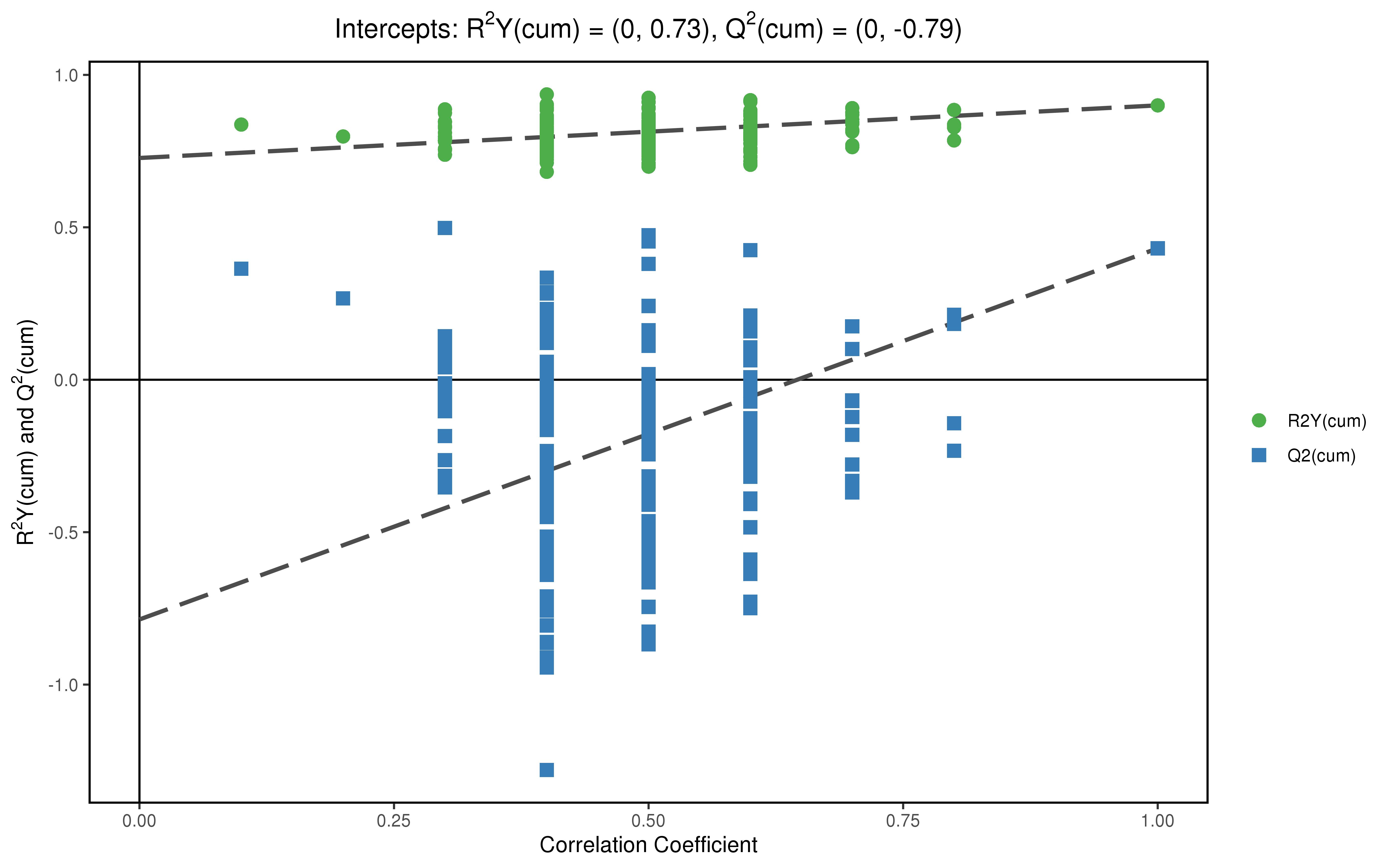

Supplement: Supplementary file 1 — Additional file 1: Fig. S1. PCA (A and B), OPLS-DA (C and D) score plots and OPLS-DA permutation plots (E and F) in faeces. A and B. PCA score plots, the abscissa PC1, and the ordinate PC2 represent the scores of the principal components ranking the first and the second, respectively, and different shapes of the scattered points represent the different groups of the samples. C and D. OPLS-DA score plots, the ordinate t[1]O represents the orthogonal principal component score, the abscissa t[1]P represents the predicted principal component score of the first principal component, and different shapes of the scattered points represent the different groups of the samples. E and F. OPLS-DA permutation plots. The abscissa correlation coefficient represents relevance. The Q2 and R2Y values reflect the model predictability and the fraction of explained variance, respectively. [file 13071_2021_4807_MOESM1_ESM.zip › Sfig1F NEG OPLS-DA permutation plot.jpg]

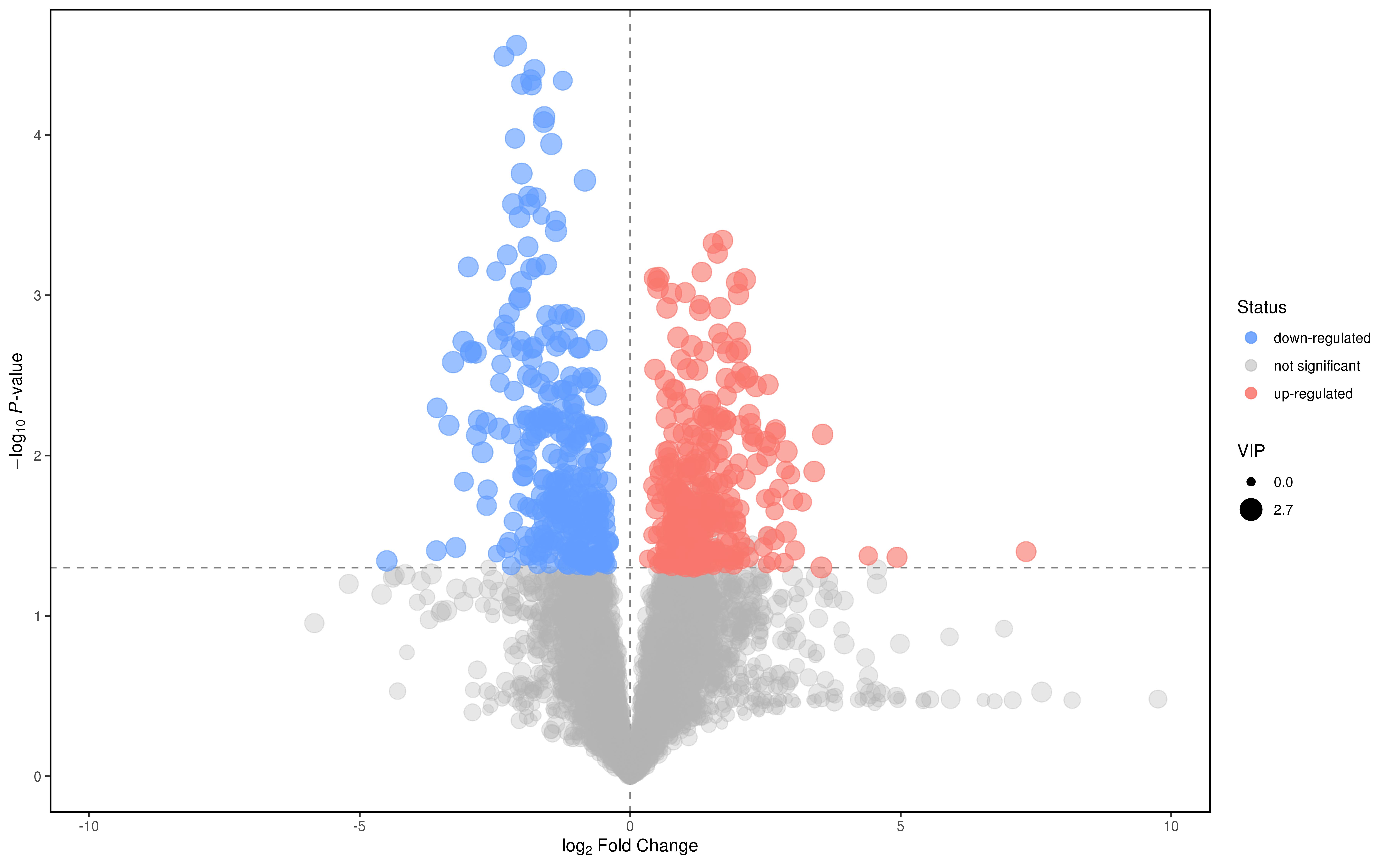

Supplement: Supplementary file 2 — Additional file 2: Fig. S2. Volcano plots in faeces (A and B). A and B. Volcano plots, each dot in the volcano map represents a metabolite, the abscissa shows the fold change value (take the logarithm of cardinal number 2), the ordinate represents the p value of Student's t test (take the negative number of base logarithm of 10), and the size of the scatter represents the VIP value of the OPLS-DA model: the larger the scatter, the greater the VIP value. [file 13071_2021_4807_MOESM2_ESM.zip › S2A volcano plot.jpg]

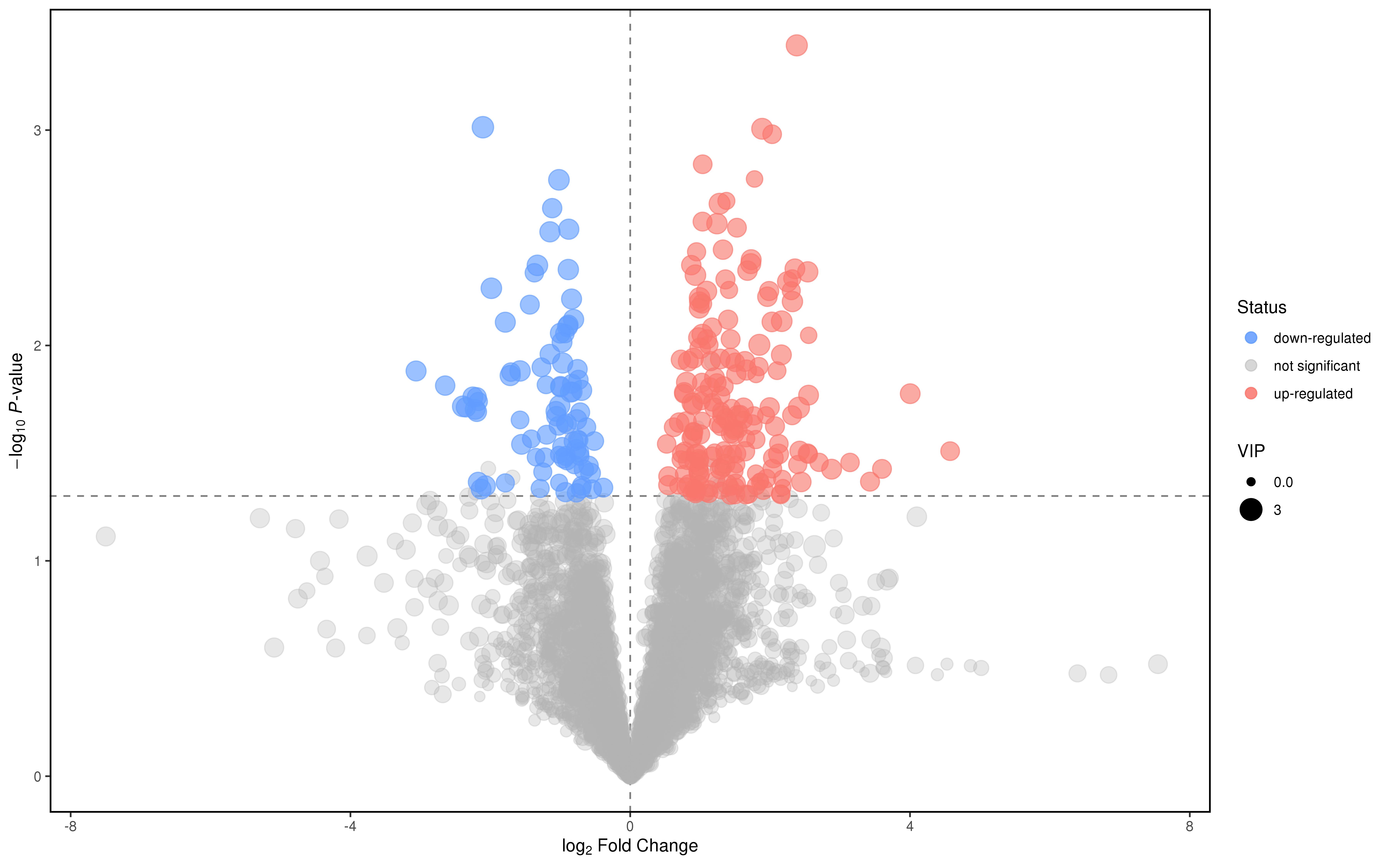

Supplement: Supplementary file 2 — Additional file 2: Fig. S2. Volcano plots in faeces (A and B). A and B. Volcano plots, each dot in the volcano map represents a metabolite, the abscissa shows the fold change value (take the logarithm of cardinal number 2), the ordinate represents the p value of Student's t test (take the negative number of base logarithm of 10), and the size of the scatter represents the VIP value of the OPLS-DA model: the larger the scatter, the greater the VIP value. [file 13071_2021_4807_MOESM2_ESM.zip › S2B volcano plot.jpg]

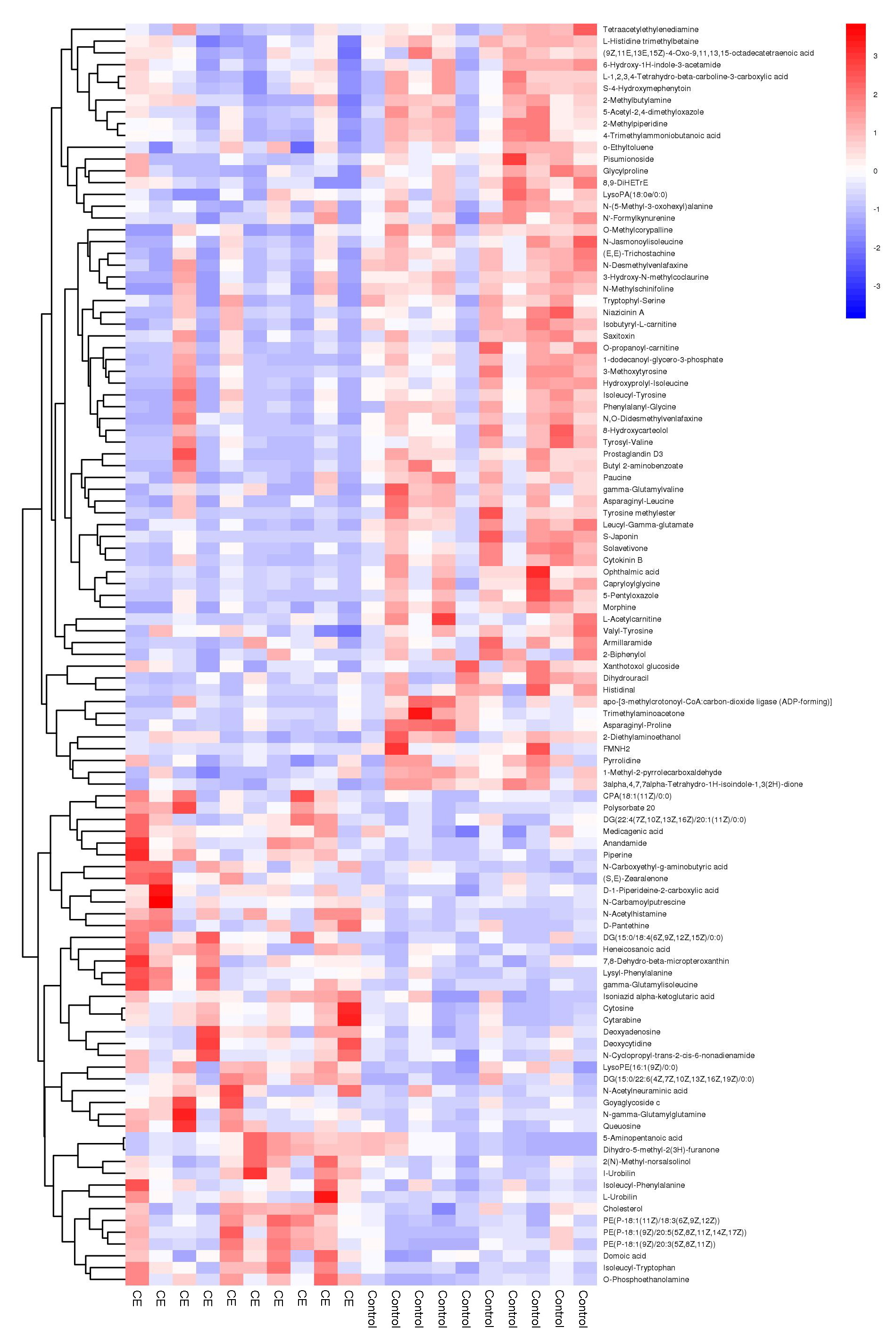

Supplement: Supplementary file 4 — Additional file 4: Fig. S3. Heat map in faeces (A and B). A and B. The abscissa indicates different experimental groups, the ordinate means different metabolites compared with the normal control group, and the square coloured blocks at different spaces represent the relative expression of metabolites at corresponding positions. [file 13071_2021_4807_MOESM4_ESM.zip › Sfig 3A heatmap.tif]

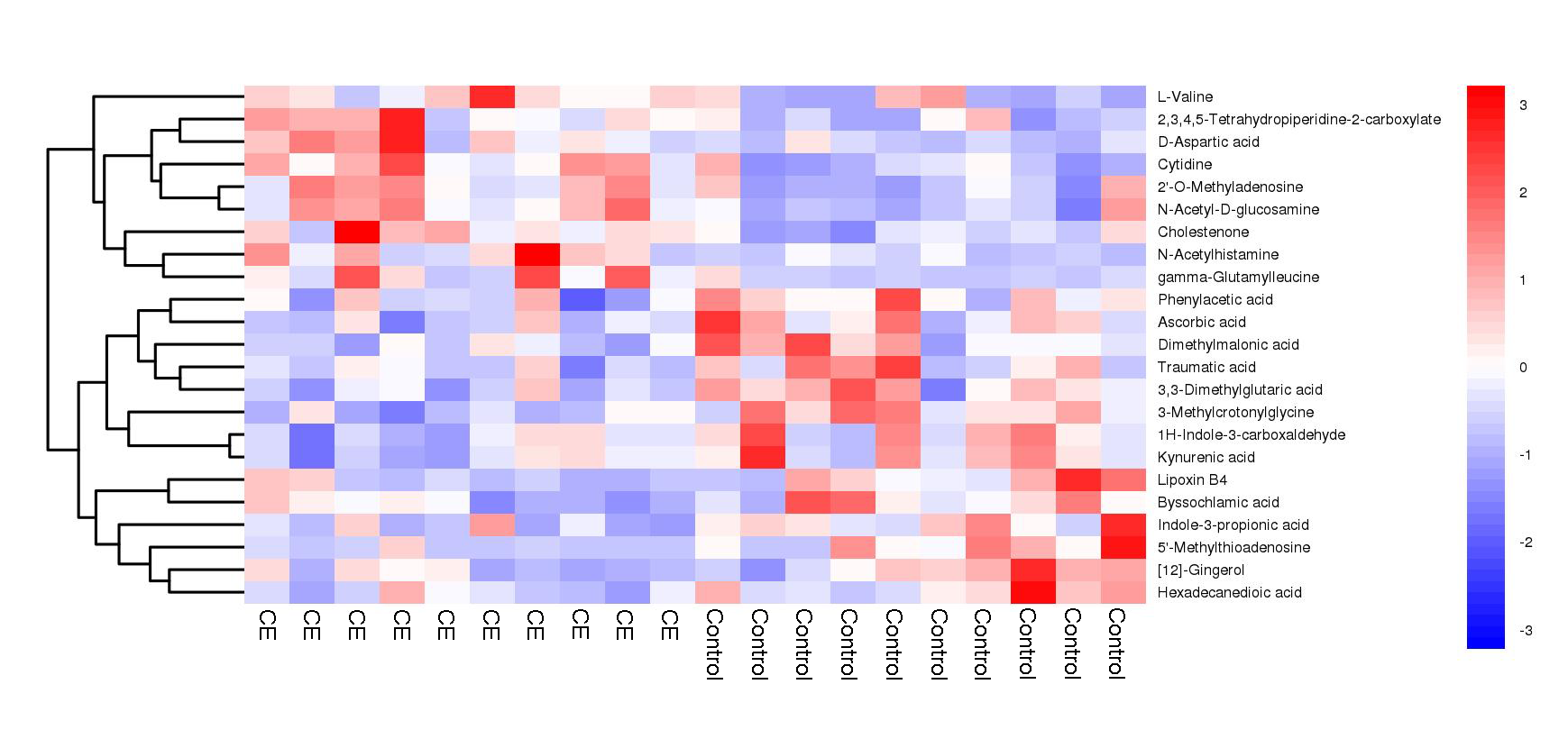

Supplement: Supplementary file 4 — Additional file 4: Fig. S3. Heat map in faeces (A and B). A and B. The abscissa indicates different experimental groups, the ordinate means different metabolites compared with the normal control group, and the square coloured blocks at different spaces represent the relative expression of metabolites at corresponding positions. [file 13071_2021_4807_MOESM4_ESM.zip › Sfig 3B heatmap.tif]

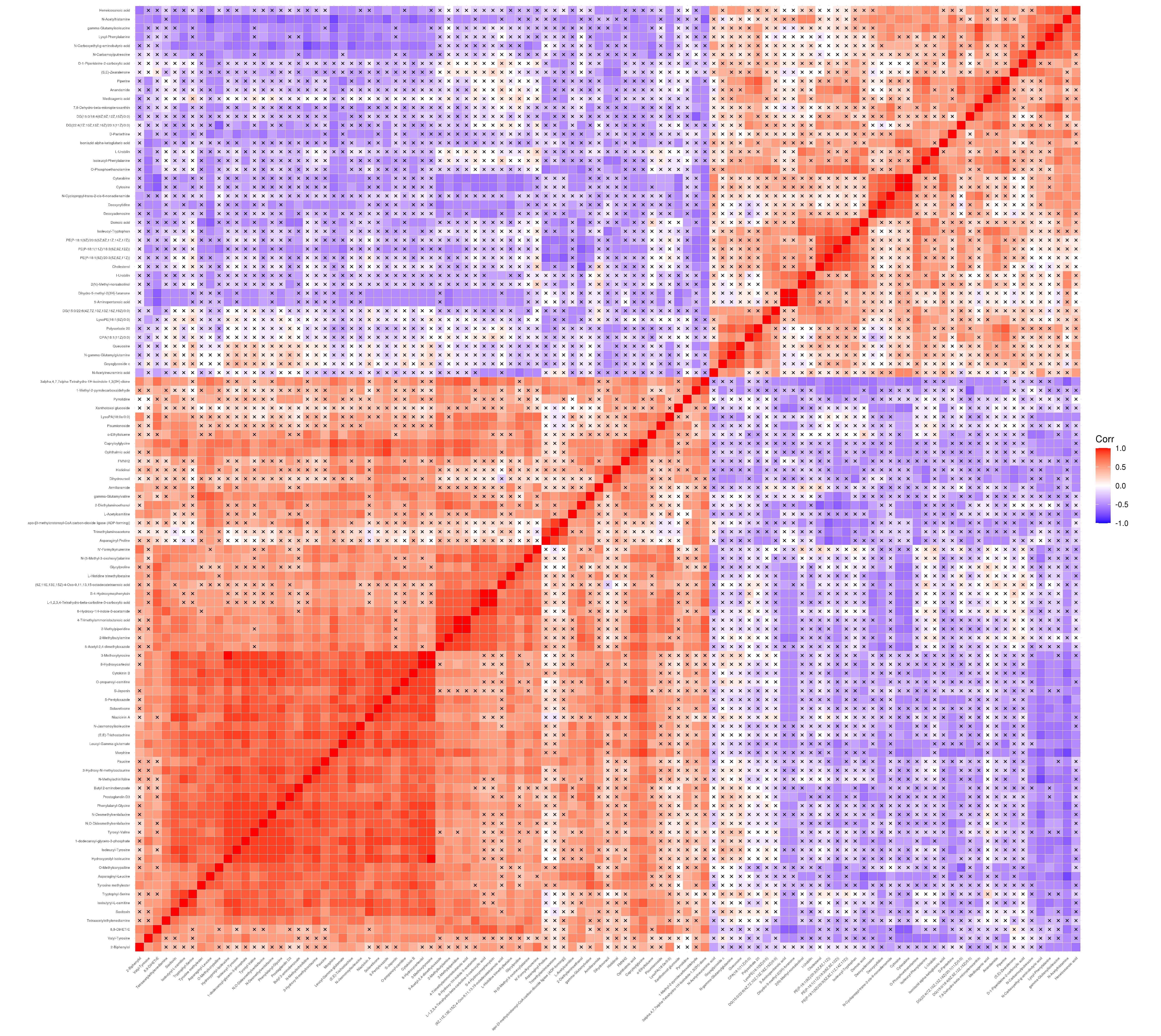

Supplement: Supplementary file 5 — Additional file 5: Fig. S4. Heat map of correlation analysis for group in faeces (A and B). A and B. The abscissa and ordinate represent the different metabolites of the group comparison. The square coloured blocks in different positions represent the correlation coefficient between the two metabolites at corresponding positions. Red shows positive correlation, blue shows negative correlation, and the darker the colour, the stronger the correlation. At the same time, the nonsignificant correlation was marked with a cross. [file 13071_2021_4807_MOESM5_ESM.zip › Sfig 4A Correlation plot.jpg]

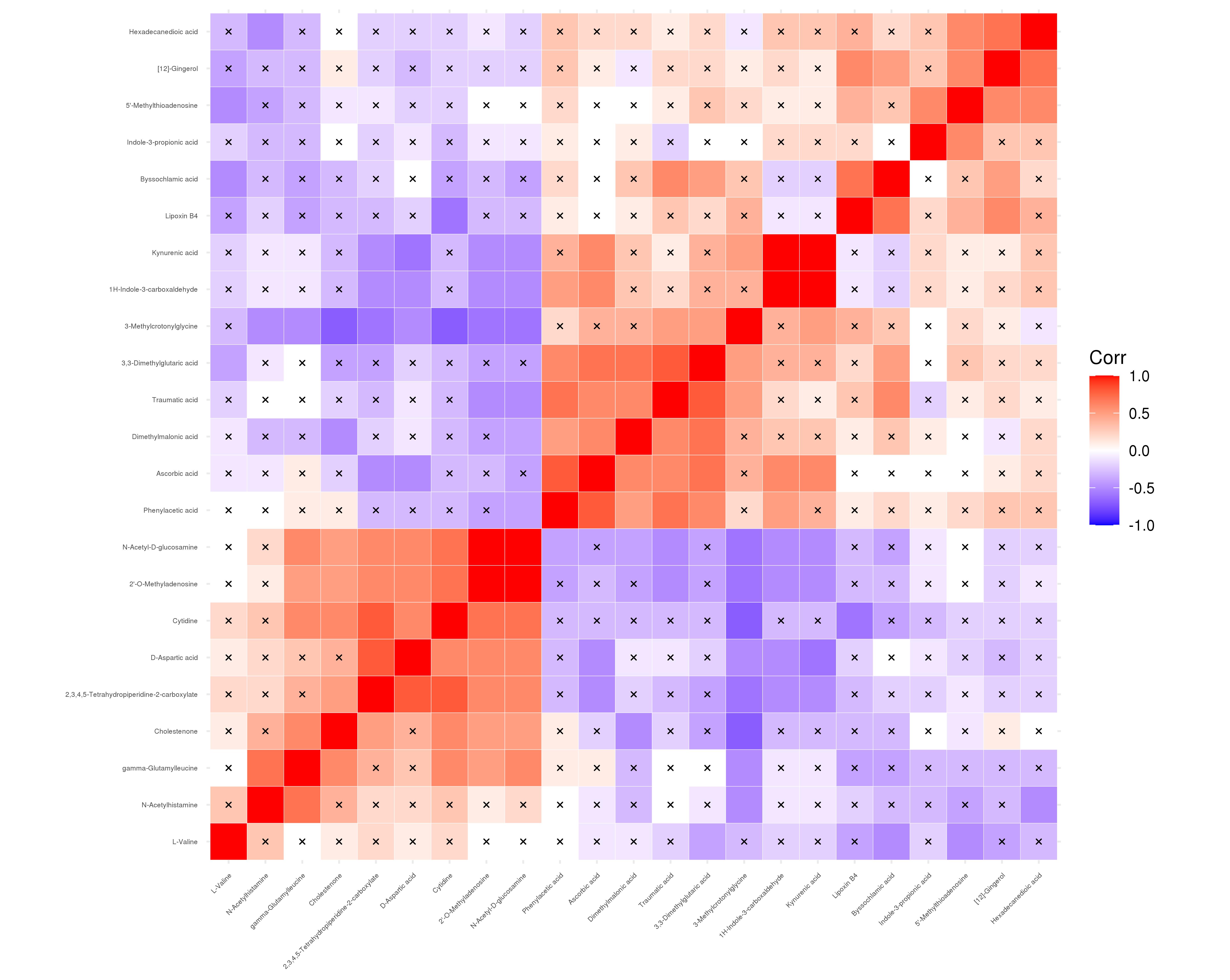

Supplement: Supplementary file 5 — Additional file 5: Fig. S4. Heat map of correlation analysis for group in faeces (A and B). A and B. The abscissa and ordinate represent the different metabolites of the group comparison. The square coloured blocks in different positions represent the correlation coefficient between the two metabolites at corresponding positions. Red shows positive correlation, blue shows negative correlation, and the darker the colour, the stronger the correlation. At the same time, the nonsignificant correlation was marked with a cross. [file 13071_2021_4807_MOESM5_ESM.zip › Sfig 4B Correlation plot.jpg]

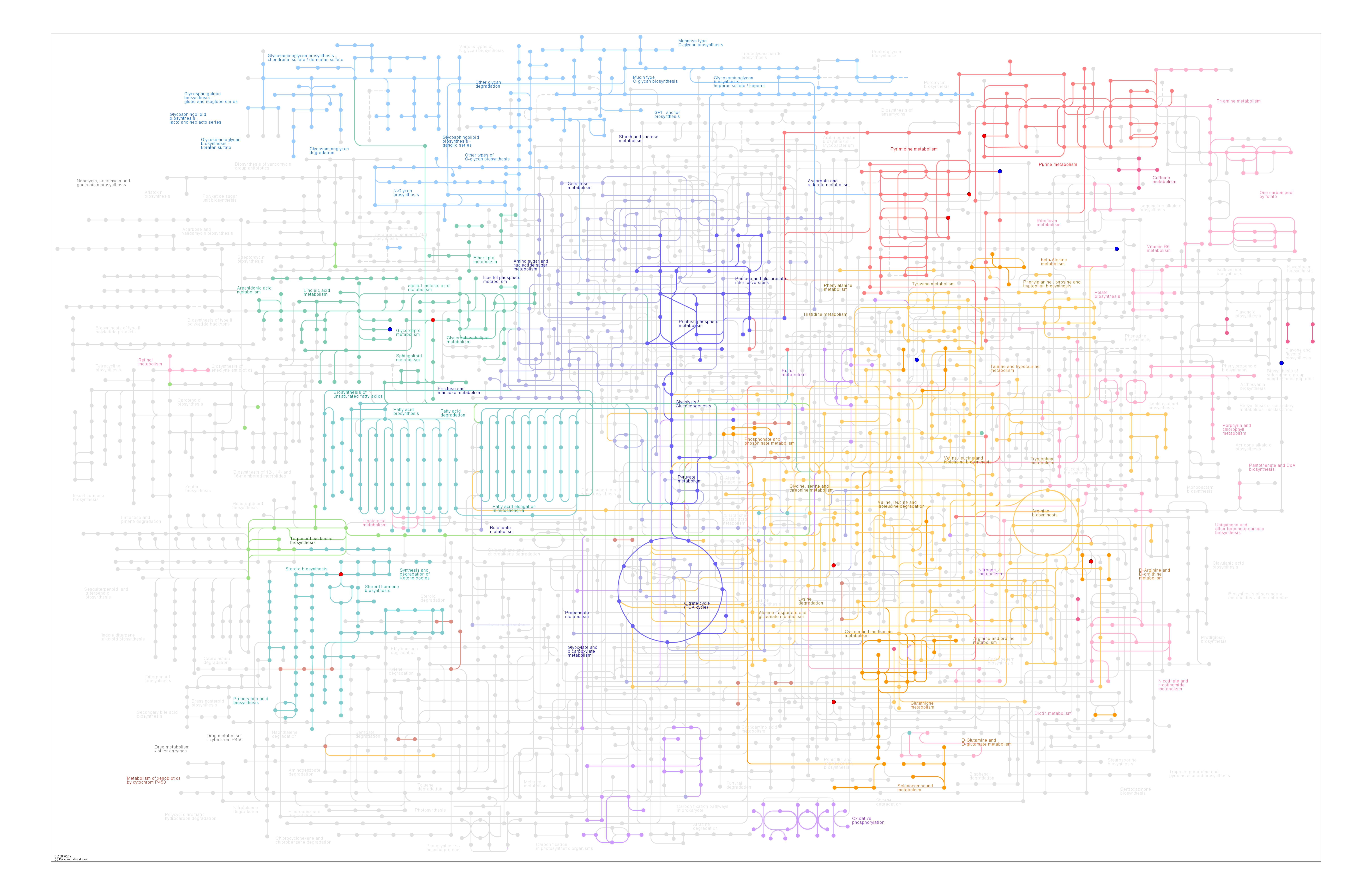

Supplement: Supplementary file 6 — Additional file 6: Fig. S5. KEGG pathways map in faeces (A and B). A and B. KEGG pathways map. The red and blue dots indicate the metabolic pathways involved by the differentially expressed metabolites. [file 13071_2021_4807_MOESM6_ESM.zip › Sfig 5A mmu01100.png]

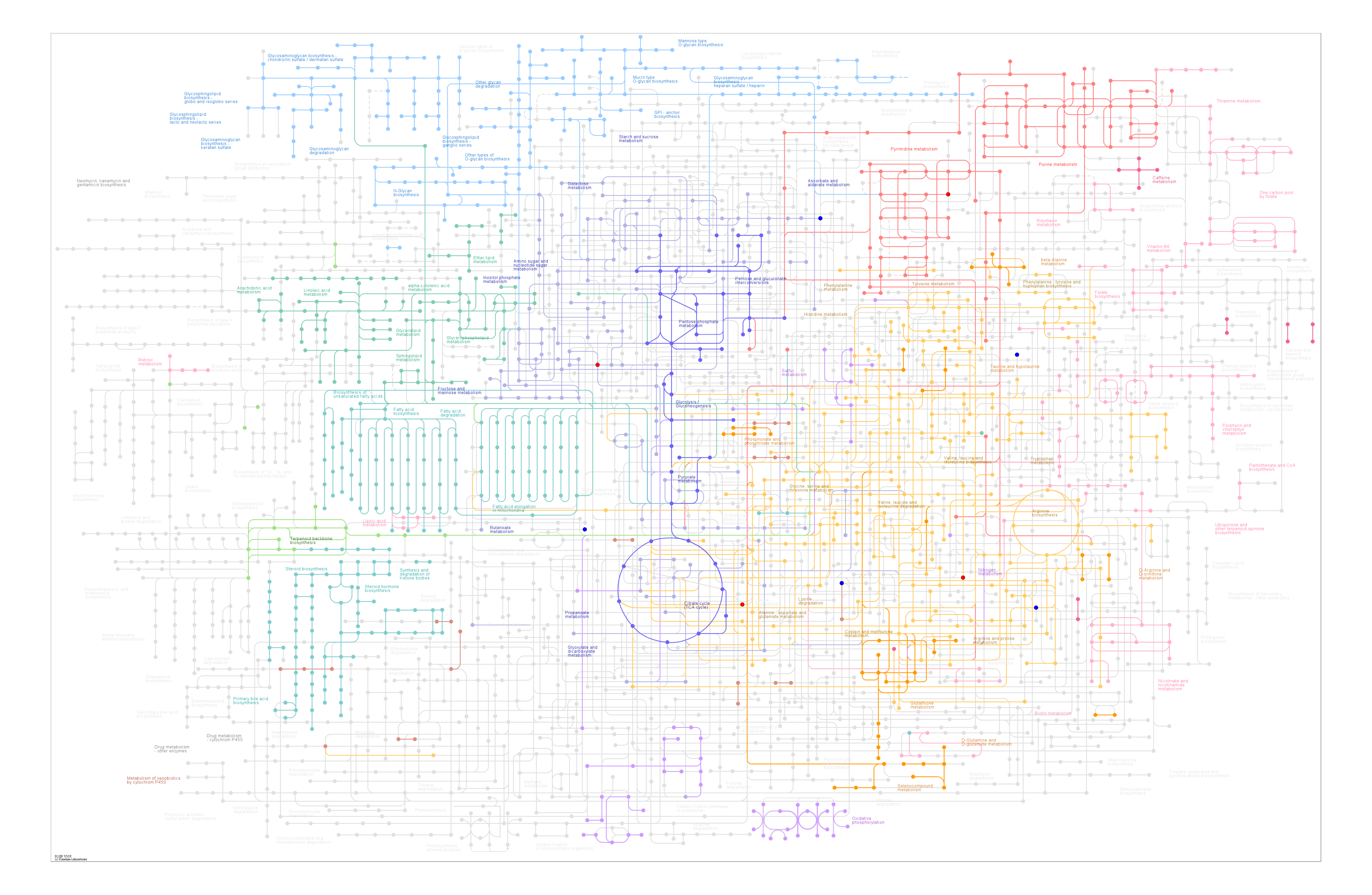

Supplement: Supplementary file 6 — Additional file 6: Fig. S5. KEGG pathways map in faeces (A and B). A and B. KEGG pathways map. The red and blue dots indicate the metabolic pathways involved by the differentially expressed metabolites. [file 13071_2021_4807_MOESM6_ESM.zip › Sfig 5B mmu01100.png]

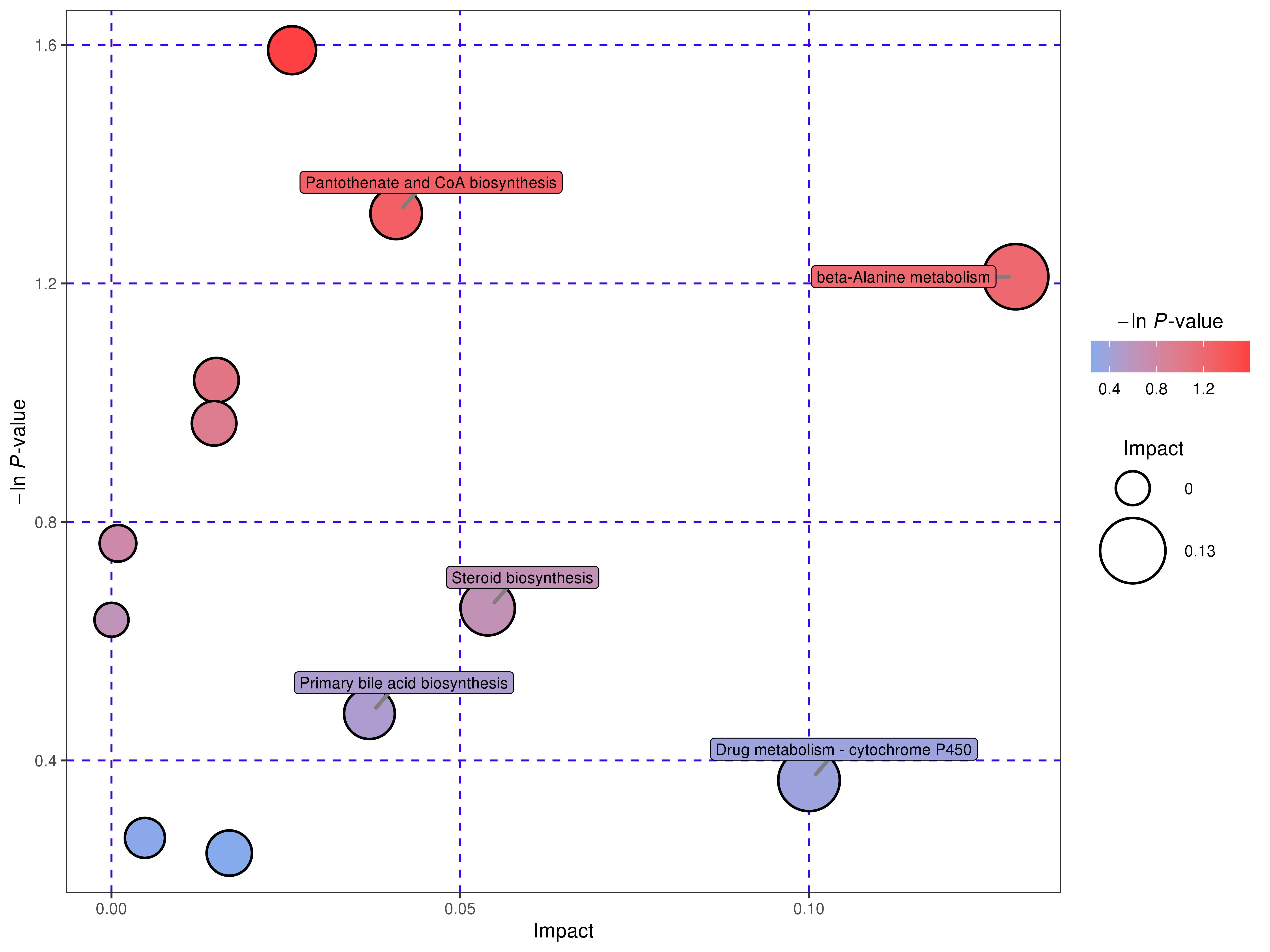

Supplement: Supplementary file 7 — Additional file 7: Fig. S6. Pathway analysis for group in faeces (A and B). A and B. Pathway analysis for group. In the bubble plots, different bubbles represent different metabolic pathway. The abscissa and the size of the bubble indicate the influence factor of the pathway in topological analysis. The larger the size, the greater the influence factors. The ordinate and the colour of the bubble show the p value of enrichment analysis (negative natural pair, i.e. - in (P)). The deeper the colour, the smaller the p value, and the more significant the enrichment degree. [file 13071_2021_4807_MOESM7_ESM.zip › Sfig 6A Bubble Plot.jpg]

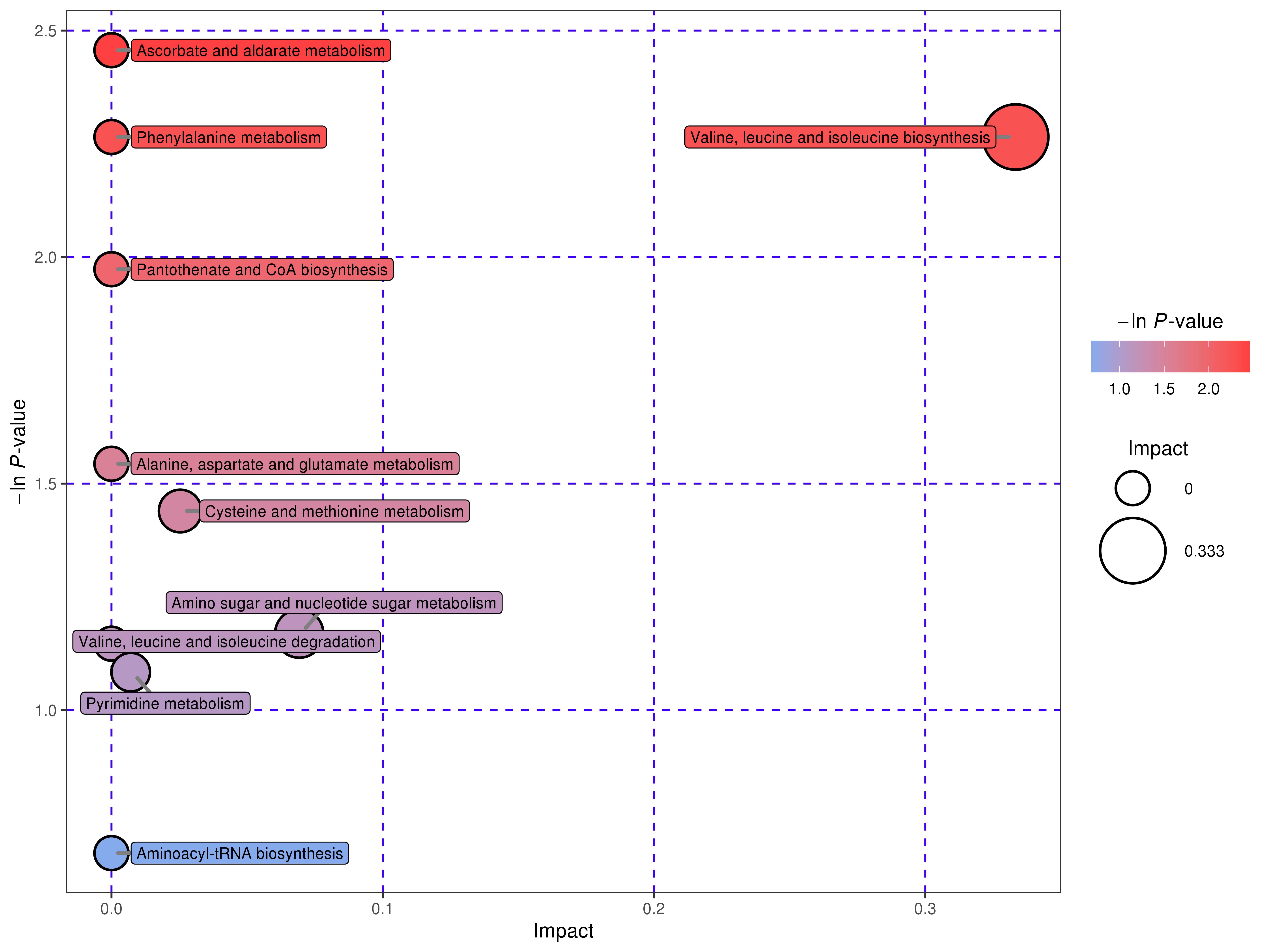

Supplement: Supplementary file 7 — Additional file 7: Fig. S6. Pathway analysis for group in faeces (A and B). A and B. Pathway analysis for group. In the bubble plots, different bubbles represent different metabolic pathway. The abscissa and the size of the bubble indicate the influence factor of the pathway in topological analysis. The larger the size, the greater the influence factors. The ordinate and the colour of the bubble show the p value of enrichment analysis (negative natural pair, i.e. - in (P)). The deeper the colour, the smaller the p value, and the more significant the enrichment degree. [file 13071_2021_4807_MOESM7_ESM.zip › Sfig 6B Bubble Plot.jpg]
